# Exploring Potential Bioactive Peptides in Fermented Bactrian Camel’s Milk and Mare’s Milk Made by Mongolian Nomads

**DOI:** 10.3390/foods9121817

**Published:** 2020-12-07

**Authors:** Khuukhenbaatar Ganzorig, Tadasu Urashima, Kenji Fukuda

**Affiliations:** 1 Department of Animal and Food Hygiene, Obihiro University of Agriculture and Veterinary Medicine, 2-11 Nishi, Inada-Cho, Obihiro, Hokkaido 080-8555, Japan; kh_ganzo@yahoo.com; 2 Department of Life and Food Sciences, Obihiro University of Agriculture and Veterinary Medicine, 2-11 Nishi, Inada-Cho, Obihiro, Hokkaido 080-8555, Japan; urashima@obihiro.ac.jp; 3 Department of Agriculture and Animal Science, Research Center for Global Agromedicine, Obihiro University of Agriculture and Veterinary Medicine, 2-11 Nishi, Inada-Cho, Obihiro, Hokkaido 080-8555, Japan

**Keywords:** *Camelus bactrianus*, casein-derived peptides, fermented milk, food functionality

## Abstract

To date, bioactive proteins and peptides from minor livestock milks and their fermented products have been scarcely reported. In Mongolia, nomads are commonly rearing five livestock animal species (i.e., cow, camel, goat, horse, and sheep) for milking and other purposes. In this study, we analyzed the peptide composition in fermented milks of Bactrian camels (*Camelus bactrianus*) and horses, produced by Mongolian nomads for self-consumption. Peptides from skimmed fermented milks were separated by ultrafiltration and reverse-phase high-performance liquid chromatography. Then, their amino acid sequences were determined by matrix-assisted laser desorption/ionization time-of-flight tandem mass spectrometry. Consequently, eleven peptides were identified in the fermented camel’s milk including four from β-casein (β-CN), three from α_s1_-CN, and two from both κ-CN and lactophorin. On the other hand, twenty-four peptides were identified in the fermented mare’s milk including nineteen from β-CN, three from α_s1_-CN, and one from both κ-CN and α_s2_-CN. According to previous reports on the bioactivities of milk-derived peptides, antibacterial and antihypertensive activities were promising in both the fermented camel’s milk and mare’s milk. In addition, potential antioxidant activity was conjectured in the fermented camel’s milk. Further investigations are currently needed to clarify the potential role of immunomodulatory peptides in the two fermented milks.

## 1. Introduction

The *Camelus* genus includes two domesticated species of camels: Dromedary camel (*Camelus dromedaries*, one-humped) and Bactrian camel (*Camelus bactrianus*, two-humped). According to statistics from the Food and Agriculture Organization (FAO), in 2018, the total population of Dromedary and Bactrian camels and available worldwide camel milk production yield were 35.8 million heads and 3.15 million tons (http://www.fao.org/faostat/en/#home). More than 75% of camel milk is assumed to be consumed by nomads as raw milk and processed dairy products. In 2018, camel milk production in Mongolia was 114,830 tons, accounting for 3.6% of the available camel milk production in the world. For the nomads living in the Gobi region of Mongolia, camels are multipurpose livestock animals, e.g., they supply milk, meat, wool, and hide as well as transportation. Because nomads consume greater quantities of Bactrian camel milk than milk from other domestic animals, they have established processing methods specialized for Bactrian camel’s milk that were inherited across generations. They believe that Bactrian camel’s milk has an anti-swelling and therapeutic effect on intestinal and kidney diseases [1]. In fact, fermented camel’s milk has been used traditionally to treat edema in pregnant women and as anti-scorbutic agent for the elderly in Mongolia.

It was reported that the general constituents of a Bactrian camel’s colostrum obtained at 2 h postpartum were 14.23% protein, 4.44% lactose, 0.27% fat, and 0.77% ash [2]. On the other hand, the same authors reported the composition of the mature milk collected at 90 d postpartum as follows: 3.55% protein, 4.24% lactose, 5.65% fat, and 0.87% ash. Similar to cow’s milk, protein in camel’s mature milk is approximately 3–4%; however, it lacks β-lactoglobulin which is the main component of whey fraction in cow’s milk. Whereas certain amounts of peptides apparently exist in milk, most bioactive peptides are encrypted in milk proteins in an inactive form, and those peptides are released by the action of proteolytic enzymes during either the fermentation process of dairy foods or digestion in the gastrointestinal tract of consumers [3,4]. Milk-derived bioactive peptides are of particular interest in food science and nutrition because they show important physiological and biological functions such as opioid-like [5], immunomodulating [6], antibacterial [7], and antihypertensive activities [8] as well as the ability to enhance calcium absorption [9]. From this point of view, several studies on the composition of dromedary camel’s milk and its fermented product have been performed [10,11]. A recent review illustrated that antihypertensive, antioxidant, and antibacterial activities had been experimentally attributed to several peptide fractions prepared from fermented dromedary camel’s milks and the proteolytic enzyme-treated milk proteins [12]. Furthermore, the identified dromedary camel’s milk-derived bioactive peptides were mostly from caseins. In addition, glycosylation-dependent cell adhesion molecule 1 (GlyCAM-1) and peptidoglycan recognition protein 1 were found as sources of angiotensin converting enzyme (ACE)-inhibitory dipeptides, VY and LY. However, knowledge pertaining to peptides occurring in fermented Bactrian camel’s milk is still scarce.

Domestication of horses (*Equus caballus*) occurred approximately 5000 years ago, and it was assumed that its purpose was mostly for transport, which had a significant contribution to the development of rural societies [13]. It is still unclear when milking horses and the production of fermented mare’s milk started, whereas fermented mare’s milk has traditionally been produced and consumed in Eurasian steppe areas since then. To date, Mongolia is known as a top producer of mare’s milk, although the amount produced is statistically unclear; it is mainly produced into a fermented mare’s milk beverage, airag. It was reported that the gross composition of mare’s milk on average was: 2.14% protein, 1.21% fat, and 6.37% lactose [14]. Fermented mare’s milk has been reported to show a positive effect on the immune system and for treatment of pulmonary tuberculosis and heart disease [15]. In general, however, information about peptides in the fermented mare’s milk is very limited. So far, the presence of antihypertensive peptides in a fermented mare’s milk, koumiss [16], and horse milk casein hydrolysate [17] has been reported. In addition, β-lactoglobulin-derived antidiabetic peptides were found in mare’s whey protein hydrolysates [18].

In this study, several peptides were isolated from fermented milks of Bactrian camels and horses using ultrafiltration and reverse-phase high-performance liquid chromatography (RP-HPLC). Amino acid sequences of the partially purified peptides were determined by matrix-assisted laser desorption/ionization time-of-flight tandem mass spectrometry (MALDI TOF-MS/MS), and two peptides were further identified by Edman degradation. The potential bioactivities of the peptides isolated from fermented Bactrian camel’s milk and mare’s milk collected in Mongolia are discussed according to their similarity to previously identified milk-derived bioactive peptides [19].

## 2. Materials and Methods

### 2.1. Reagents

A Centriprep YM-3 filter device was purchased from Millipore (Billerica, MA, USA). Acetonitrile (ACN), trifluoroacetic acid (TFA), and protease inhibitor cocktail for mammals were purchased from Sigma–Aldrich (St. Louis, MI, USA). Buffer solutions for the RP-HPLC were filtered through a 0.2 μm membrane filter from Millipore prior to use. Peptide calibration standard II (1–3 kDa) used for calibration of the MALDI TOF-MS, MALDI target plate, and α-cyano-4-hydroxycinnamic acid (α-CHCA) were purchased from Bruker Daltonik GmbH (Bremen, Germany). The HPLC system was a JASCO (Tokyo, Japan) and equipped with a PU-2089 pump, UV 2075 detector, MX 2080-32 dynamic mixer, and a CO-8020 column oven. The TSK gel ODS-80Ts (0.46 × 25 cm) column was from Tosoh Co. (Tokyo, Japan). All other chemicals were of analytical grade.

### 2.2. Milk Samples

Fermented Camel’s milk was produced from September 2015 to April 2016, and fermented mare’s milk from August to October 2016. Approximately 500 mL each of Bactrian camel’s fermented milks were collected from 3 different herders: two herders near Hamriin hiid, Ulaanbadrakh soum, Dornogovi aimag and one herder in Dalanzadgad soum, Umnugovi aimag in Mongolia on 16 October 2016. Approximately 500 mL each of the mare’s fermented milk were also collected from 3 herders located in a narrow region, 3–5 km apart from each other, in Adaatsag soum, Dundgovi aimag, Mongolia, on 2 October 2016. The numbers of milked animals in the herds ranged from 10–20 and 15–24 heads for camels and horses, respectively, at the time of sampling. It was confirmed that both the fermented camel’s milk and mare’s milk were free from contamination of milks derived from other livestock animals via investigation with the nomads who made them. All samples were collected and stored independently in sterile containers and kept at 4 °C in cooling boxes during transport (3 h at maximum) from the farms to the State Central Veterinary Laboratory in Ulaanbaatar and kept at −30 °C until frozen transport to Japan.

### 2.3. Peptide Purification

Each of the fermented camel’s milk and mare’s milk samples (100 mL) was mixed with 1 mL of the protease inhibitor cocktail for mammals and defatted by centrifugation at 500× *g* for 10 min at 20 °C. The fat and cells were removed and then a supernatant as a whey was centrifuged again at the same condition to remove residual cream. Residual caseins and insoluble compounds were removed by centrifugation at 26,900× *g* for 60 min at 4 °C. The supernatant, namely, acid whey, was subjected to ultrafiltration at 4 °C using the Centriprep YM-3 (nominal molecular weight limit (NMWL) 3000). Membrane permeate was lyophilized and dissolved in 0.1% of TFA at 80 mg/mL concentration. A 100 μL aliquot of membrane permeate was injected into the ODS-80Ts column (0.46 × 25 cm), pre-equilibrated with 0.1% TFA, and connected to the HPLC system. After 5 min of static flow with 100% solvent A (0.1% TFA in water), elution was performed by a linear gradient from 0% to 50% solvent B (0.1% TFA in ACN) for 90 min at a flow rate of 1.0 mL/min and at 30 °C. The eluent was monitored by an ultraviolet detector at the wavelength of 214 nm. Eluted peptide fractions were collected manually, concentrated by rotary and vacuum evaporation, and lyophilized. The lyophilized peptides were dissolved in 50 µL of 0.1% TFA and stored at −20 °C until used.

### 2.4. Mass Spectrometry

The peptide solution was desalted using ZipTip C18 pipette tips (Millipore, Bedford, MA, USA) according to the manufacturer’s instructions. A 1.0 μL aliquot of the desalted peptide solution and an equal volume of 10 mg/mL of α-CHCA saturated in 0.1% TFA/ACN (2:1, v/v) were mixed, and 1.0 μL of the mixture was loaded on a target plate (MTP 384 target plate ground steel T F, Bruker, Bremen, Germany). After the solvent dried, the target plate was mounted in an AutoflexII TOF/TOF mass spectrometer (Bruker, Bremen, Germany). Mass spectra were obtained using the pre-installed method, RP_1-3kDa (a reflector positive ion mode optimized to the mass range of 1–3 kDa). Peptide calibration standard II was used as an external mass calibrant. The acquired spectra were statistically analyzed using Flexanalysis 2.0 software (Bruker, Bremen, Germany). Amino acid sequences of the peptides were determined by fragmentation analysis of MALDI-generated ions using a technique of LIFT-TOF/TOF MS (MS/MS). The mass list of fragment ions was searched using the BioTools 3.0 interface (Bruker, Bremen, Germany) connected to the Mascot search engine [20]. In brief, other Mammalia were selected as taxonomy, and either MSDB, SwissProt, or NCBInr was chosen as a database (http://www.matrixscience.com/index.html). None of the fixed modifications were selected, but varied settings of the enzyme, variable modifications, peptide tolerance (±50–250 ppm), and MS/MS tolerance (±0.5–1.0 Da) were tested. Other settings were used as default. Peptides were identified only when their probability based Mowse scores showed statistical significance. In addition, amino acid sequence similarity was analyzed using protein BLAST interface at National Center for Biotechnology Information (NCBI) (https://blast.ncbi.nlm.nih.gov/).

### 2.5. N-Terminal Sequence Analysis

N-terminal amino acid sequences of the peptides were determined by the Edman degradation method [21] and following HPLC separation of phenylthiohydantoin derivatives of amino acids using the Procise 492 HPLC system (PerkinElmer, Waltham, MA, USA) at the Instrumental Analysis Division, Equipment Management Center, Creative Research Institution, Hokkaido University.

## 3. Results

### 3.1. Peptide Profiles in the Fermented Milks Analyzed by RP-HPLC

**Figure 1:**
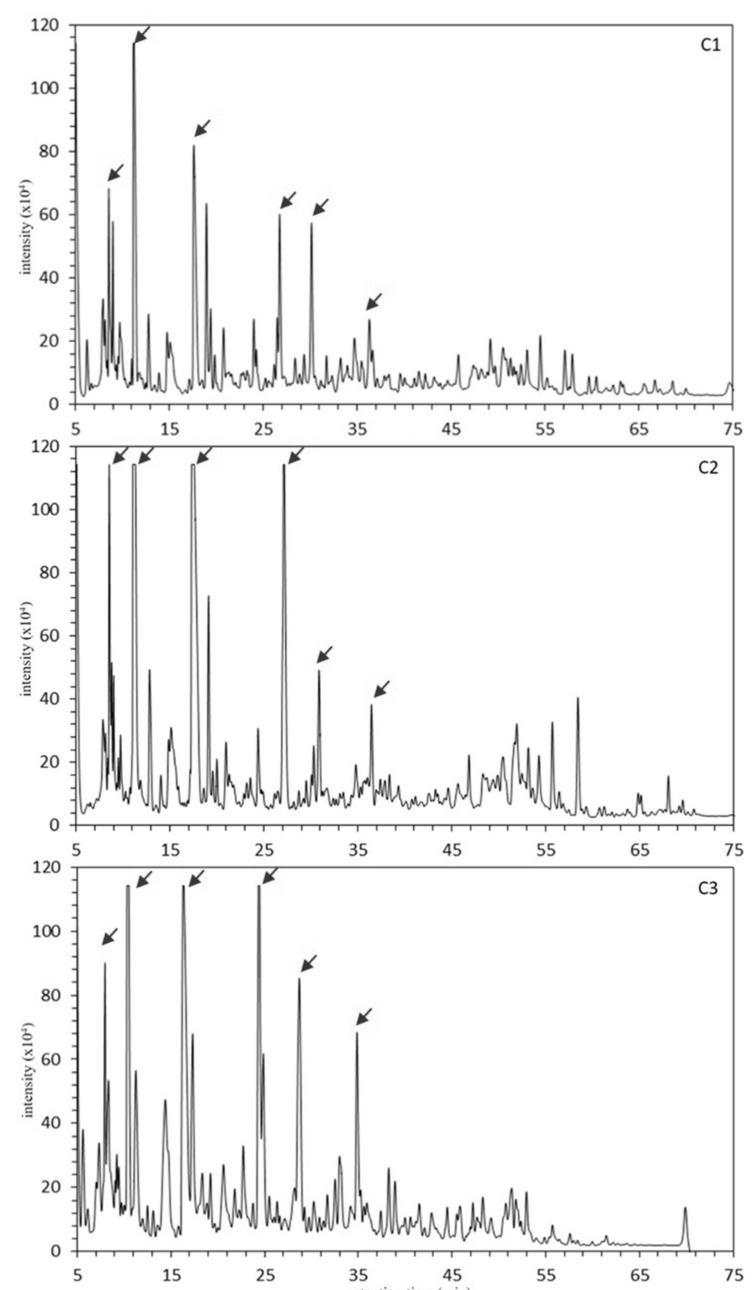
The reverse-phase high-performance liquid chromatograms (RP-HPLC) of ultrafiltrates (NMWL 3000) of three fermented Bactrian camel’s milks obtained from different herders (C1–C3). Arrows indicate fractions manually collected for MS/MS peptide identification. The peak IDs were given in accordance with their retention time, e.g., the arrow indicating the left-most peak’s ID in C3 is C3–1.

**Figure 2:**
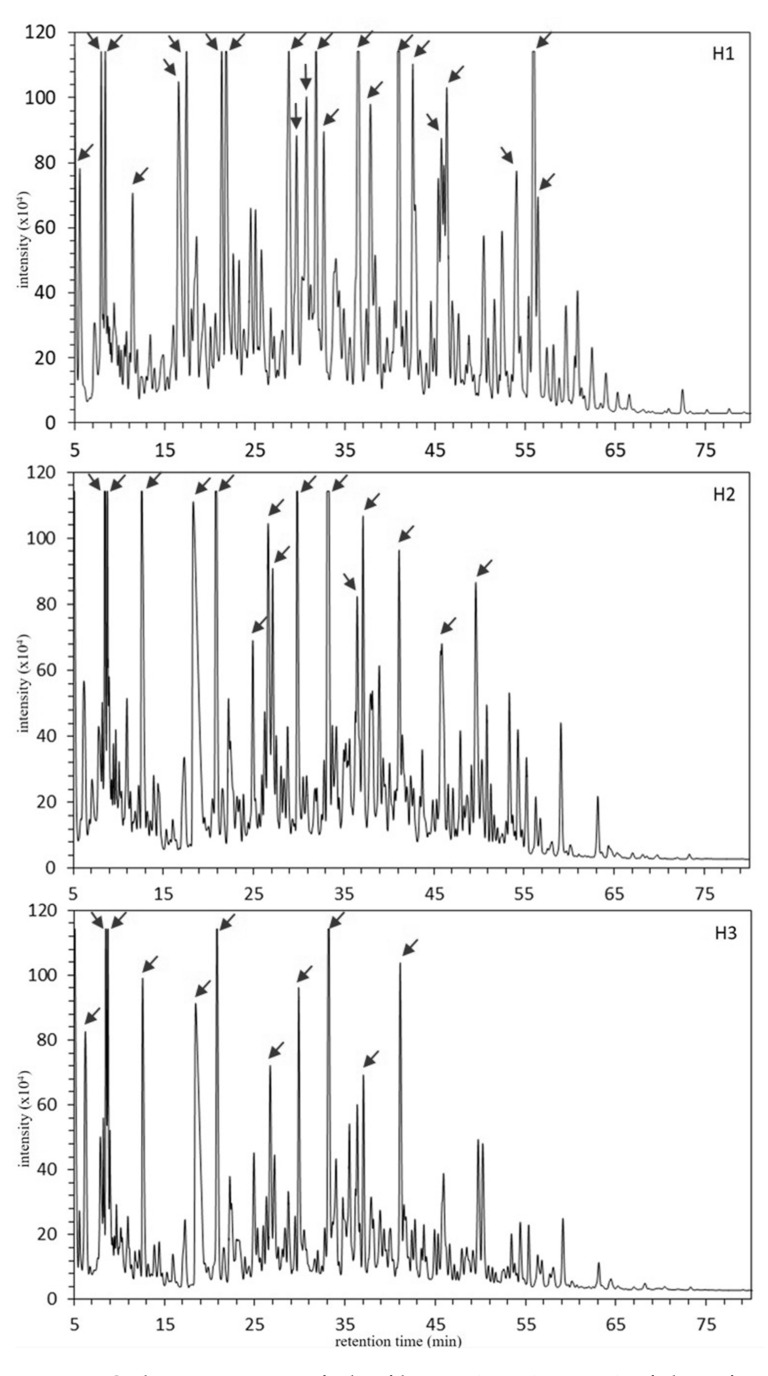
The RP-HPLC chromatograms of ultrafiltrates (NMWL 3000) of three fermented mare’s milks obtained from different herders. Arrows indicate fractions manually collected for MS/MS peptide identification. Arrows indicate fractions manually collected for MS/MS peptide identification. The peak IDs were given in accordance with their retention time, e.g., the arrow indicating the left-most peak’s ID in H1 is H1–1.

Low molecular weight fractions (< kDa) prepared from fermented camel’s milk and mare’s milk using the Centriprep YM-3 were further separated with RP-HPLC. Fermented camel’s milk and mare’s milk showed distinct chromatograms with the RP-HPLC as shown in Figure 1 and Figure 2. Ten to eleven major peaks and a number of minor peaks were found in three fermented camel’s milks (C1, C2, and C3 in Figure 1). On the other hand, eleven to twenty-two major peaks (22 peaks for H1, 15 peaks for H2, and 11 peaks for H3) and plenty of minor peaks were observed in the fermented mare’s milk (H1, H2, and H3 in Figure 2). No peaks appeared in all chromatograms after 75 min to the end of elution; hence, chromatograms after 75 or 80 min of elution were omitted in Figure 1 and Figure 2. Consequently, C3 and H1, which showed the highest number of major peaks among camel and mare fermented milks, respectively, were selected as representatives for further fractionation and identification of peptides. In total, 33 peaks, of which 11 peaks from C3 (numbered from C3–1 to C3–11 according to their elution order) and 22 peaks from H1 (numbered from H1–1 to H1–22 according to their elution order) were manually collected and then analyzed by MALDI TOF-MS/MS.

### 3.2. Peptide Analysis by MALDI TOF-MS/MS

**Figure 3:**
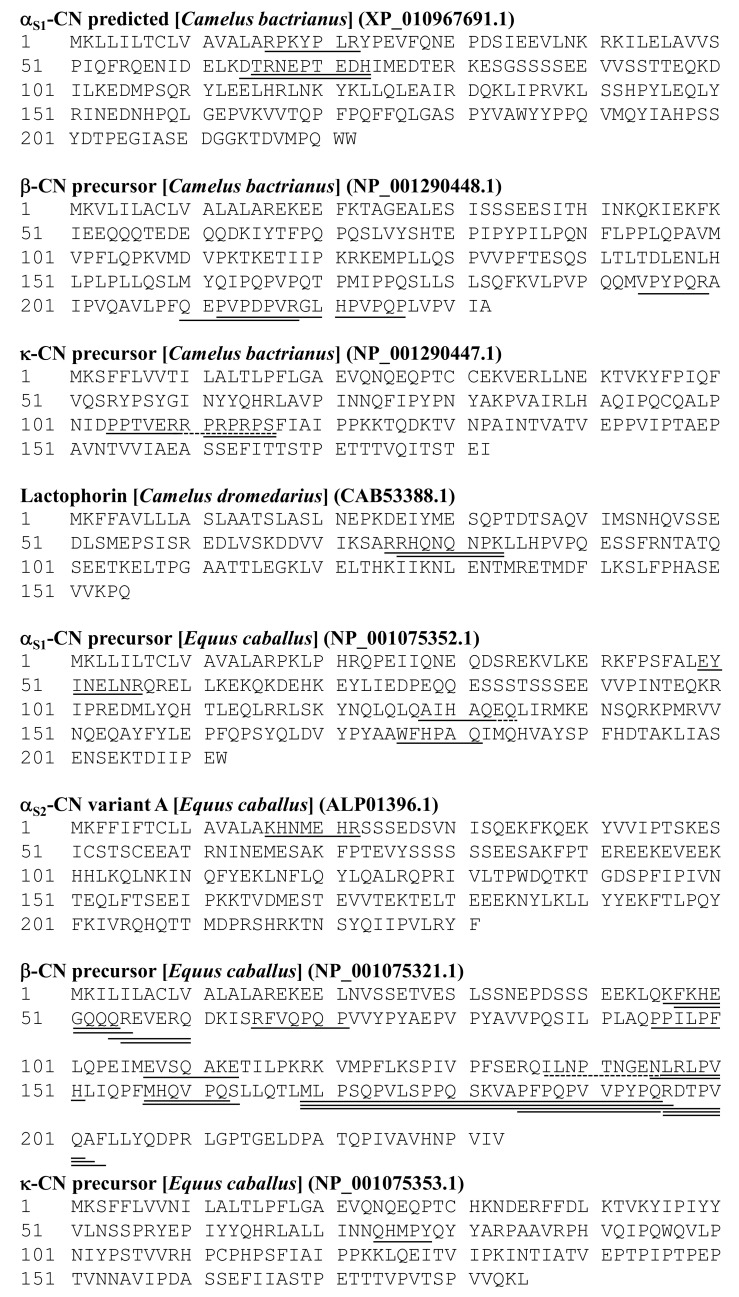
Location of the identified peptides in their mother proteins. The peptides identified in this study are indicated by underlines. The NCBI reference numbers of the mother proteins are indicated in the parentheses. The numbers present at the left-most of the column indicate the number of amino acid residues that started from the N-terminal methionine of the precursors. Dotted lines indicate sequence inconsistency between the identified peptide and the mother protein used as reference, probably due to the presence of genetic variants.

The results of the peptide analysis on the 11 peaks separated from the fermented camel’s milk, C3, are summarized in Figure 3 and Table 1. Two lactophorin-derived peptides, ^76^HQNQNPK^83^ and R^75^RHQNQNPK^83^, were identified in peaks C3–3 and C3–4, respectively (Figures S1 and S2). Two α_s1_-CN-derived peptides, T^65^RNEPTEDH^73^ and D^64^TRNEPTEDH^73^, were found in peak C3–5 (Figure S3), and another α_s1_-CN-derived peptide, R^16^PKYPLR^22^, was in peak C3–10 (Figure S7). In total, four β-CN-derived peptides were identified. Among them, two peptides, H^221^PVPQP^226^ and P^212^VPDPVRGL^220^, were found in peak C3–8 (Figure S5). Another two β-CN-derived peptides, V^194^PYPQR^199^ and Q^210^EPVPDPVR^218^, were found in peaks C3–9 and C3–11, respectively (Figures S6 and S8). Moreover, the N-terminal glutamine residue in the latter peptides had a possibility of being pyroglutamylated, which gave 17 Da a smaller molecular mass than the unmodified peptide. A κ-CN-derived peptide, R^110^PRPRPS^116^, was found in peaks C3–6 (Figure S4) and C3–7 (data not shown). Another κ-CN-derived peptide, P^104^PTVERPARNRHD^116^, was found in peak C3–9, but MS/MS analysis of the latter part was incomplete (Figure S6). Amino acid sequence of κ-CN-derived peptide P^104^PTVERRPRPRPS^116^ at the same position was reported in dromedary camel [22], but its molecular mass was 1543.870, which was slightly higher than what we detected; therefore, unknown mutations or post-translational modifications may occur in the peptides isolated from the fermented Bactrian camel’s milk. Despite successful analysis of IRIPV in peaks C3–1 and C3–2, NLRLPV and HLLQPF in peak C3–3, and NNASHNGNNSAPI in peak C3–8, their origins were not assigned to camel milk proteins and remain unknown.

The results of the peptide analysis of the 22 peaks separated from fermented mare‘s milk H1 are summarized in Figure 3 and Table 2. These identified peptides were fragments of either α_s1_-, α_s2_-, β-, or κ-CN. Three α_s1_-CN-derived peptides, A^128^IHAQRK^134^, E^49^YINELNR^56^, and W^176^FHPAQ^181^, were found in peaks H1–3, H1–13, and H1–14, respectively (Figure S11, S17 and S18). One α_2_-CN-derived peptide, K^16^HNMEHR^22^, was present in peak H1–2 (Figure S10). The majority of the peptides found in fermented mare‘s milk H1 derived from β-CN. Two β-CN-derived peptides, E^107^VSQAKE^113^ and R^55^EVERQ^60^, were found in H1–1 (Figure S9). A β-CN-derived peptide, Q^54^REVERQ^60^, and a form of ammonium ion loss from the N-terminal of the peptide were found in H1–3 (Figure S11). Two similar β-CN-derived peptides, K^46^FKHEGQQQ^54^ and F^47^KHEGQQQR^55^, were present both in H1–4 and H1–6 (Figures S12 and S13). A β-CN-derived peptide, M^157^HQVPQS^163^, was found in H1–7 and H1–8 in which another β-CN-derived peptide, R^196^DTPVQA^202^, was present (Figures S14 and S15). In H1–10, a β-CN-derived peptide, R^65^FVQPQP^71^, was detected (Figure S16). Two β-CN-derived peptides, R^196^DTPVQAF^203^ and P^185^FPQPVVPYPQ^195^, were found in H1–14 (Figure S18). Moreover, two β-CN-derived peptides, N^145^LRLPV^150^ and K^137^LIPTPNGRSLRLPVH^151^, were present in H1–19 (Figure S19). The asparagine residue in the latter peptide seemed to be deamidated. The sequence variation found in this sequence may be due to the presence of a genetic variant or unidentified modifications; this requires further investigation. Similar β-CN-derived peptides were observed, such as M^169^LPSQPVLSPPQSKVAPFPQPVPYPQR^196^ in H1–20, M^169^LPSQPVLSPPQSKVAPFPQPVPYPQRDTPVQ^201^ in H1–20 and H1–22, and M^169^LPSQPVLSPPQSKVAPFPQPVPYPQ^195^ in H1–21 (Figures S20–S22). In addition, a short β-CN-derived peptide, P^95^PILPF^100^, was identified in H1–22 (Figure S22). One κ-CN-derived peptide, Q^74^HMPY^78^, was apparent in H1–13 (Figure S17). In total, 24 CN-derived peptides were identified in the ultra-filtrates of the fermented mare‘s milk. Although amino acid sequences were successfully deduced for QGRRGKP in H1–9 and KVPMPPH in H1–14 (Figure S18) by MS/MS analysis, their origins remain unclear. Amino acid sequences of two peptides in H1–3 were further identified to be A^128^IHAQRK^134^ and Q^54^REVERQ^60^ by the N-terminal sequence analysis in order to confirm the accuracy of the peptide identification by using MS/MS.

H1—fermented camel’s milk sample No 1; n.d.—not determined. Two peptides, A^135^IHAQRK^141^ and Q^54^REVERQ^60^, found in the H1–3 fraction and which had sequences that were confirmed by Edman degradation are indicated in bold. Numbers in superscript indicate the peptide position in the original protein sequence (see Figure 3).

## 4. Discussion

This study aimed to provide basic information on the peptide profiles of fermented Bactrian camel’s milk and mare’s milk produced by Mongolian nomads. The RP-HPLC chromatograms of low molecular fractions (< kDa) of the three individual fermented camel’s milks (C1, C2, and C3) showed a lower number of major peaks compared to those of the fermented mare’s milks (H1, H2, and H3) (Figure 1 and Figure 2). Among C1, C2, and C3, a high similarity of the peak profiles was observed between C1 and C2 (collected from two herders near Hamriin hiid, Ulaanbadrakh soum, Dornogovi aimag), and those were partially different from C3 (collected from one herder in Dalanzadgad soum, Umnugovi aimag). This may be partly due to the different individual caseins’ polymorphism, although further investigation is required. Another possible reason for the similarity in the peptide profiles of C1 and C2 is that they shared common microbiota to a large extent, probably owing to intercommunion among the nomads living in the area. Although H1, H2, and H3 were collected in the same area, Adaatsag soum, Dundgovi aimag, their RP-HPLC chromatograms were largely different, probably reflecting the unique microbiota in each of them. It is controversial that different microbiota could provide similar peptide profiles when the same milk material and similar manufacturing process were applied for the production of fermented milk products. Parallel analyses on the microbiota and the peptide profile using the same fermented milk sample should provide a clue on this issue.

In this study, 11 and 24 peptides were identified in the fermented camel’s milk C3 and the fermented mare’s milk H1, respectively. A high number of unidentified peptides could arise from presence of unknown post-translational modifications in their mother proteins. Peptides originated from caseins were dominant in both C3 and H1. Especially, a variety of β-CN-derived peptides accounted for the majority of the identified peptides due to the high abundance of β-CN (45–65% of total caseins) in camel or mare milks [14,33]. The most specific feature of peptides found in the fermented camel’s milk C3 was the presence of κ-CN- and lactophorin-derived peptides (Figure 1 and Table 1). The κ-CN-derived peptide, R^110^PRPRPS^116^, could be aligned using protein BLAST with a C-terminal region of bovine para-κ-CN HPHPHLS, which is known to be produced by chymosin treatment during cheese manufacturing [34]. This part of camel’s κ-CN is in the proximity of the chymosin cleavage site [35]. To date, no health-promoting effect has been reported for this peptide, in contrast to glycomacropeptide, which is the counterpart to the para-κ-CN in the chymosin hydrolysate of κ-CN. Bovine lactophorin and its N- and C-terminal truncated variant (f3-135) are known as proteose-peptone component 5 [36] and GlyCAM-1 [37], respectively. On the other hand, the lactophorin-derived peptides, R^75^RHQNQNPK^83^ and R^76^HQNQNPK^83^, found in this study were embedded in the middle part of mature lactophorin. Ibrahim et al. [38] recently found two lactophorin-derived peptides, E^113^NTMRETMDFLKSLF^127^ and A^79^TTLEGKLVEL^89^, in pepsin-digested camel milk whey hydrolysates that confer tolerance against H_2_O_2_-induced oxidative stress to yeast cells. The biological significance of the lactophorin-derived peptides found in the fermented camel’s milk in this study remains to be elucidated. One α_2_-CN-derived peptide, K^16^HNMEHR^22^, was characteristically found in fermented mare’s milk H1, but its bioactivity also remains unclear.

To date, a number of α- and β-CN-derived peptides are known to exhibit ACE inhibitory activity (e.g., β-casokinins), antibacterial, antioxidant, immunomodulatory, mineral binding, and opioid agonists (e.g., β-casomorphins) activities [39,40,41]. Several biological activities were noticed as having potential for the peptides identified in this study, although further biological characterizations are required. Regarding peptides showing potential ACE-inhibitory activity, three β-CN-derived peptides, H^221^PVPQP^226^, V^194^PYPQR^199^, and Q^210^EPVPDPVR^218^—which are similar to known ACE-inhibitory peptides PVPQP from human β-CN [23], AVPYPQR from bovine β-CN [27], and YQEPVLGPVR from bovine β-CN [28]—were found in the fermented camel’s milk C3. On the other hand, two β-CN-derived peptides, R^196^DTPVQAF^203^ and N^145^LRLPV^150^—which were similar to the reported ACE-inhibitory peptides RDMPIQAF from bovine β-CN [32] and NLHLP from human β-CN [23]—were found in the fermented mare’s milk H1. Due to the fact of their high sequence similarities (>75% amino acid sequence identity), a potential ACE-inhibitory activity from fermented camel’s milk and mare’s milk may be considered likely.

It has been reported that a mixture of peptides, including α_s1_-CN R^16^PKYP^20^, β-CN Q^192^MVPYPQR^199^, β-CN V^206^LPFQEPVPDPVRG^219^, and β-CN L^221^HPVPQP^226^, isolated from fermented camel’s milk, showed antibacterial activity against *Escherichia coli*, *Staphylococcus aureus*, *Staphylococcus faecalis*, and *Staphylococcus dysenteria* [24]. Similar peptides, α_s1_-CN R^16^PKYPLR^22^ and β-CN V^194^PYPQR^199^, Q^210^EPVPDPVR^218^, H^221^PVPQP^226^, were found in this study in the fermented camel’s milk C3; hence, it is highly possible that these peptides could show antibacterial activity. In addition, V^194^PYPQR^199^, found in fermented camel’s milk, is very similar to bovine β-CN-derived AVPYPQR, which has been reported as an antimicrobial peptide [25]. In fact, antibacterial activity of C3 ultrafiltrate has been observed toward *Shigella sonnei* and *Salmonella* Typhymurium, but the antibacterial substances are still to be identified (unpublished data). The peptide M^157^HQVPQ^162^, found in mare’s fermented milk H1, was similar to MHQPPQPL, which has been identified in goat β-CN as a peptide showing antimicrobial [30] and dipeptidyl peptidase (DPP)-IV inhibitory activities [31].

Antioxidant activity could be expected to come from a β-CN-derived peptide V^194^PYPQR^199^ found in the fermented camel’s milk C3, owing to the high sequence similarity to AVPYPQR, which has been reported as an antioxidant peptide derived from bovine β-CN [26]. Similar human β-CN-derived peptides, VPYPQ, QVVPYPQ, and PYPQ, have also been reported as antioxidant peptides [42,43]. Recently, three antioxidant peptides, RLDGQGRPRVWLGR, TPDNIDIWLGGIAEPQVKR, and VAYSDDGENWTEYRDQGAVEGK, have been newly found in Bactrian camel’s milk hydrolysates prepared by the action of several proteolytic enzymes, including trypsin, pepsin, alcalase, and papain [44]; however, we could not find any similar peptides to the three antioxidant peptides in our experiment.

It is evidenced that casein-derived peptides, produced by the action of digestive enzymes, such as trypsin, pepsin, and chymosin, can exhibit immunomodulatory activities [45]. In this study, no immunomodulative peptides identical to those which have already been found in bovine CN-derived peptides were found. Only one peptide, Q^210^EPVPDPVR, was found in fermented camel’s milk fraction C3–11, as being similar (77.8% amino acid sequence identity) to a bovine β-CN derived peptide, Y^208^QEPVLGPVR^217^, which showed stimulatory activity against human peripheral blood lymphocytes [29]. Therefore, further exploration of immunomodulative peptides in Mongolian fermented milks and functional characterization of such potential bioactive peptides should be performed. Finally, it should be stressed that the peptide analysis was performed with just one sample each of fermented camel’s milk and mare’s milk, and not in a quantitative manner in this study; hence, further investigations are needed.

## 5. Conclusions

Variations in peptides (<3000 Da) in Bactrian fermented camel’s milk and mare’s milk made by Mongolian nomads were partially illustrated in this study. It was confirmed that such traditional fermented milks were certainly attractive sources of bioactive peptides. Our results suggest that the presence of antihypertensive and antipathogenic peptides should be promising in Mongolian fermented camel’s milk and mare’s milk. In addition, the presence of antioxidant and antidiabetic peptides are likely in the fermented camel’s milk and mare’s milk, respectively. However, further investigation is needed to confirm the presence of immunomodulative peptides in the fermented milks. Moreover, further studies should be required for demonstrating the biological activities of the identified peptides in this study.

## Figures and Tables

**Table 1:** Identified peptides in the fermented camel’s milk C3.

Peak ID	Observed *m/z* by MS	Theoretical Mass *	Sequence Estimated by MS/MS *	Origin	Potential Bioactivity [Reference]
C3–1	597.363	596.400	IRIPV	n.d.	
C3–2	597.321	596.400	IRIPV	n.d.	
C3–3	711.386	710.440	NLRLPV	n.d.	
	754.362	753.420	HLLQPF	n.d.	
	1021.522	1020.520	R^76^HQNQNPK^83^	Lactophorin	
	1232.775	n.d.	n.d.	n.d.	
C3–4	1335.659	n.d.	n.d.	n.d.	
	1177.576	1176.620	R^75^RHQNQNPK^83^	Lactophorin	
C3–5	1098.615	1097.470	T^65^RNEPTEDH^73^	α_s1_-CN	
	1213.633	1212.500	D^64^TRNEPTEDH^73^	α_s1_-CN	
	1798.079	n.d.	n.d.	n.d.	
C3–6	865.548	864.500	R^110^PRPRPS^116^	k-CN	
C3–7	865.548	864.500	R^110^PRPRPS^116^	k-CN	
C3–8	674.370	673.350	H^221^PVPQP^226^	β-CN	ACE inhibitory [23] Antimicrobial [24]
	966.588	948.540	P^212^VPDPVRGL^220^	β-CN	
	1309.692	1308.580	NNASHNGNNSAPI	n.d.	
C3–9	759.415	758.410	V^194^PYPQR^199^	β-CN	Antimicrobial [24,25] Antioxidant [26] ACE inhibitory [27]
	1544.925	1543.800	P^104^PTVERPARNRHD^116^	k-CN	
C3–10	929.574	928.560	R^16^PKYPLR^22^	α_s1_-CN	Antimicrobial [24]
C3–11	1019.540	1035.530	Q^210^ ** EPVPDPVR^218^	β-CN	ACE inhibitory [28] Antimicrobial [24] Immunomodulative [29]
	1076.616	n.d.	n.d.	n.d.	

* Expected according to the corresponding sequence of *Camelus dromedaries.* ** N-terminal glutamine residue was likely to be pyroglutamylated (–16 Da). ACE—angiotensin converting enzyme; C3—fermented camel’s milk sample No 3; n.d.—not determined. Numbers in superscript indicate the peptide position in the original protein sequence (see Figure 3).

**Table 2:** Identified peptides in the fermented mare’s milk H1.

Peak ID	Observed *m/z* by MS	Theoretical Mass *	Sequence Estimated by MS/MS *	Origin	Potential Bioactivity
H1–1	790.394	789.390	E^107^VSQAKE^113^	β-CN	
	816.432	815.420	R^55^EVERQ^60^	β-CN	
H1–2	951.458	950.450	K^16^HNMEHR^22^	α_s2_-CN	
	1206.619	n.d.	n.d.	n.d.	
H1–3	823.490	822.480	**A** ^128^ **IHAQRK** ^134^	α_s1_-CN	
	927.470	944.020	**Q** ^54^ **REVERQ ^60^	β-CN	
	944.491	943.480	Q^54^REVERQ^60^	β-CN	
H1–4	1129.575	1128.570	K^46^FKHEGQQQ^54^	β-CN	
	1157.581	1156.570	F^47^KHEGQQQR^55^	β-CN	
H1–5	872.090	n.d.	n.d.	n.d.	
	1241.665	n.d.	n.d.	n.d.	
H1–6	1129.575	1128.570	K^46^FKHEGQQQ^54^	β-CN	
	1157.581	1156.570	F^47^KHEGQQQR^55^	β-CN	
H1–7	826.388	825.380	M^157^HQVPQS^163^	β-CN	
	1024.578	n.d.	n.d.	n.d.	
H1–8	739.356	738.350	M^157^HQVPQ^162^	β-CN	Antimicrobial [30] DPP-IV inhibitory [31]
	786.410	785.400	R^196^DTPVQA^202^	β-CN	
	851.517	n.d.	n.d.	n.d.	
	1396.762	n.d.	n.d.	n.d.	
H1–9	727.519	n.d.	n.d.	n.d.	
	2062.080	797.460	QGRRGKP	n.d.	
	1255.721	n.d.	n.d.	n.d.	
H1–10	871.484	870.470	R^65^FVQPQP^71^	β-CN	
H1–11	871.425	n.d.	n.d.	n.d.	
H1–12	1046.504	n.d.	n.d.	n.d.	
	1232.561	n.d.	n.d.	n.d.	
H1–13	506.406	n.d.	n.d.	n.d.	
	675.222	674.280	Q^74^HMPY^78^	κ-CN	
	697.237	n.d.	n.d.	n.d.	
	713.227	n.d.	n.d.	n.d.	
	866.317	n.d.	n.d.	n.d.	
	1050.508	1049.510	E^49^YINELNR^56^	α_s1_-CN	
	1171.640	n.d.	n.d.	n.d.	
	1398.713	n.d.	n.d.	n.d.	
	1980.967	n.d.	n.d.	n.d.	
	2262.040	n.d.	n.d.	n.d.	
H1–14	785.358	784.370	W^176^FHPAQ^181^	α_s1_-CN	
	805.487	804.430	KVPMPPH	n.d.	
	933.512	932.470	R^196^DTPVQAF^203^	β-CN	ACE inhibitory [32]
	1195.652	1267.660	P^185^FPQPVVPYPQ^195^	β-CN	
	1611.978	n.d.	n.d.	n.d.	
	2067.045	n.d.	n.d.	n.d.	
H1–15	734.443	n.d.	n.d.	n.d.	
	1021.563	n.d.	n.d.	n.d.	
	1460.719	n.d.	n.d.	n.d.	
H1–16	1618.056	n.d.	n.d.	n.d.	
	1954.955	n.d.	n.d.	n.d.	
H1–17	1442.653	n.d.	n.d.	n.d.	
	2069.022	n.d.	n.d.	n.d.	
	2085.990	n.d.	n.d.	n.d.	
H1–18	1798.163	n.d.	n.d.	n.d.	
	1815.285	n.d.	n.d.	n.d.	
H1–19	711.451	710.440	N^145^LRLPV^150^	β-CN	ACE inhibitory [23]
	1797.000	1797.070	K^137^LIPTPN***GRSLRLPVH^151^	β-CN	
H1–20	3084.609	3083.660	M^169^LPSQPVLSPPQSKVAPFPQPVPYPQR^196^	β-CN	
	3624.809	3623.920	M^169^LPSQPVLSPPQSKVAPFPQPVPYPQRDTPVQ^201^	β-CN	
H1–21	2928.835	2927.560	M^169^LPSQPVLSPPQSKVAPFPQPVPYPQ^195^	β-CN	
H1–22	683.468	682.410	P^95^PILPF^100^	β-CN	
	716.920	n.d.	n.d.	n.d.	
	3652.638	3651.951	M^169^LPSQPVLSPPQSKVAPFPQPVPYPQRDTPVQ^201^	β-CN	

* Expected according to the corresponding sequence of*Equus caballus*. ** Ammonium ion loss seems to have occurred at the N-terminal glutamine residue. *** Deamidation (−0.9840 Da) seems to have occurred on the asparagine residue. DPP-IV, dipeptidyl peptidase IV.

## Supplementary Materials

The following are available online at https://www.mdpi.com/2304-8158/9/12/1817/s1, Figure S1: MS spectrum of C3–3 (A) and MS/MS identification of a peak of *m/z* 1021.522 (B), Figure S2: MS spectrum of C3–4 (A) and MS/MS identification of a peak of *m/z* 1177.576 (B), Figure S3: MS spectrum of C3–5 (A) and MS/MS identification of two peaks of *m/z* 1098.615 (B) and 1213.633 (C), Figure S4: MS spectrum of C3–6 (A) and MS/MS identification of a peak of *m/z* 865.548 (B), Figure S5: MS spectrum of C3–8 (A) and MS/MS identification of two peaks of *m/z* 674.370 (B) and 966.588 (C), Figure S6: MS spectrum of C3–9 (A) and MS/MS identification of two peaks of *m/z* 759.415 (B) and 1544.925 (C), Figure S7: MS spectrum of C3–10 (A) and MS/MS identification of a peak of *m/z* 929.574 (B), Figure S8: MS spectrum of C3–11 (A) and MS/MS identification of a peak of *m/z* 1019.540 (B), Figure S9: MS spectrum of H1–1 (A) and MS/MS identification of two peaks of *m/z* 816.432 (B) and 790.394 (C), Figure S10: MS spectrum of H1–2 (A) and MS/MS identification of a peak of *m/z* 951.458 (B), Figure S11: MS spectrum of H1–3 (A) and MS/MS identification of tree peaks of *m/z* 823.490 (B), 944.491 (C), and 927.470 (D), Figure S12: MS spectrum of H1–4 (A) and MS/MS identification of two peaks of *m/z* 1129.575 (B) and 1157.581 (C), Figure S13: MS spectrum of H1–6 (A) and MS/MS identification of two peaks of *m/z* 1129.575 (B) and 1157.581 (C), Figure S14: MS spectrum of H1–7 (A) and MS/MS identification of a peak of *m/z* 826.388 (B), Figure S15: MS spectrum of H1–8 (A) and MS/MS identification of two peaks of *m/z* 739.356 (B) and 786.410 (C), Figure S16: MS spectrum of H1–10 (A) and MS/MS identification of a peak of *m/z* 871.484 (B), Figure S17: MS spectrum of H1–13 (A) and MS/MS identification of two peaks of *m/z* 675.222 (B) and 1050.508 (C), Figure S18: MS spectrum of H1–14 (A) and MS/MS identification of three peaks of *m/z* 785.358 (B), 933.512 (C), and 1195.652 (D), Figure S19: MS spectrum of H1–19 (A) and MS/MS identification of two peaks of *m/z* 711.451 (B) and 1797.000 (C), Figure S20: MS spectrum of H1–20 (A) and MS/MS identification of two peaks of *m/z* 3084.609 (B) and 3624.809 (C), Figure S21: MS spectrum of H1–21 (A) and MS/MS identification of a peak of *m/z* 2928.835 (B), Figure S22: MS spectrum of H1–22 (A) and MS/MS identification of two peaks of *m/z* 683.468 (B) and 3652.638 (C).
