# Supplementary material for: Exploring Potential Bioactive Peptides in Fermented Bactrian Camel’s Milk and Mare’s Milk Made by Mongolian Nomads
Source: Foods. 2020 Dec 7;9(12):1817. doi: 10.3390/foods9121817 (PMC7762409; doi:10.3390/foods9121817)
Supplement: Supplementary file 1 [file foods-09-01817-s001.pdf]

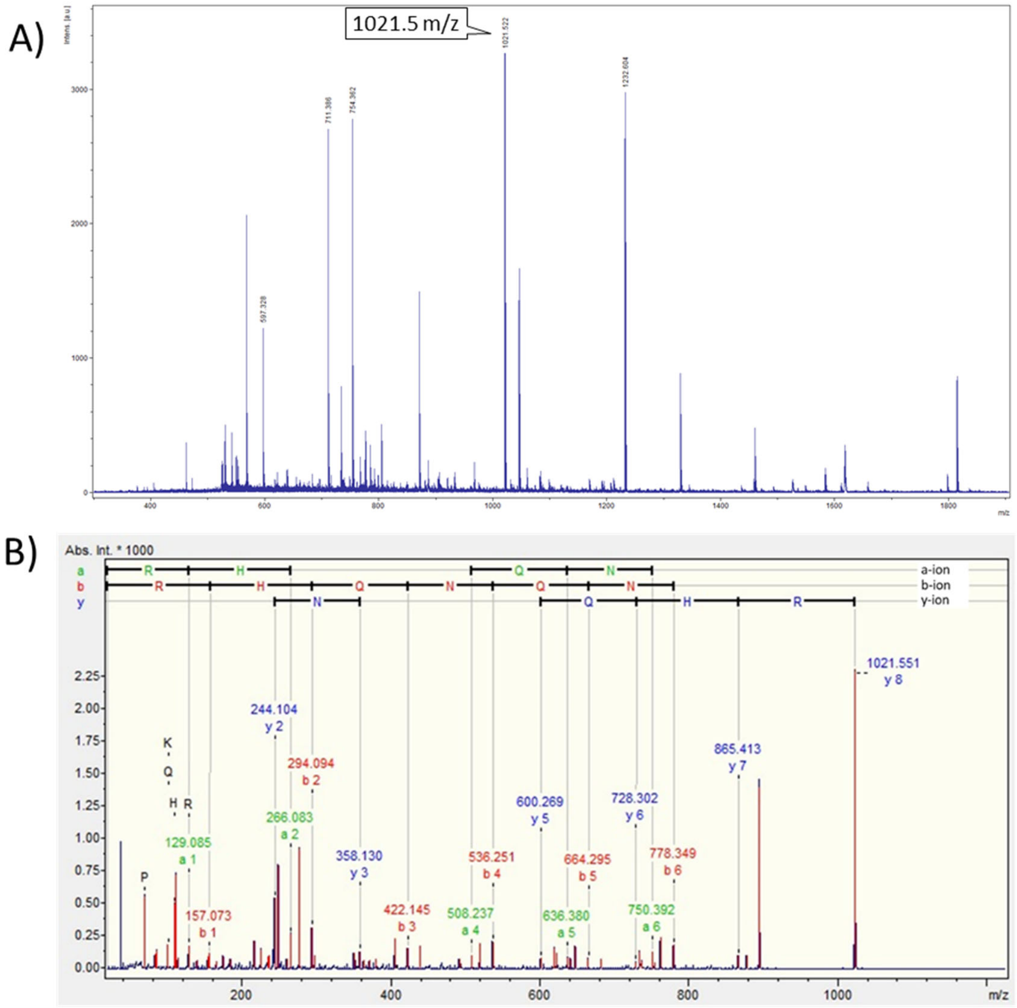


**Figure S1.** MS spectrum of C3-3 (**A**) and MS/MS identification of a peak of *m*/*z* 1021.522 (**B**).

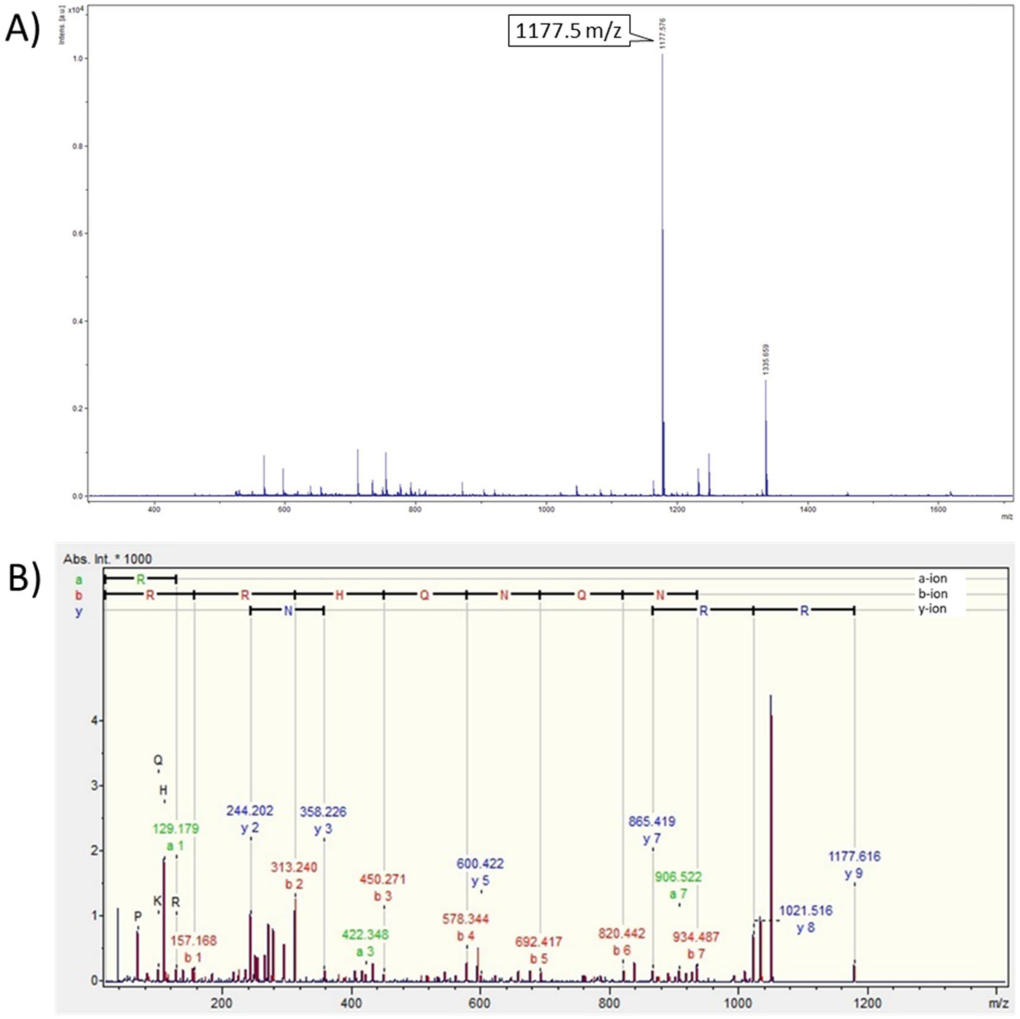


**Figure S2.** MS spectrum of C3-4 (**A**) and MS/MS identification of a peak of *m/z* 1177.576 (**B**).

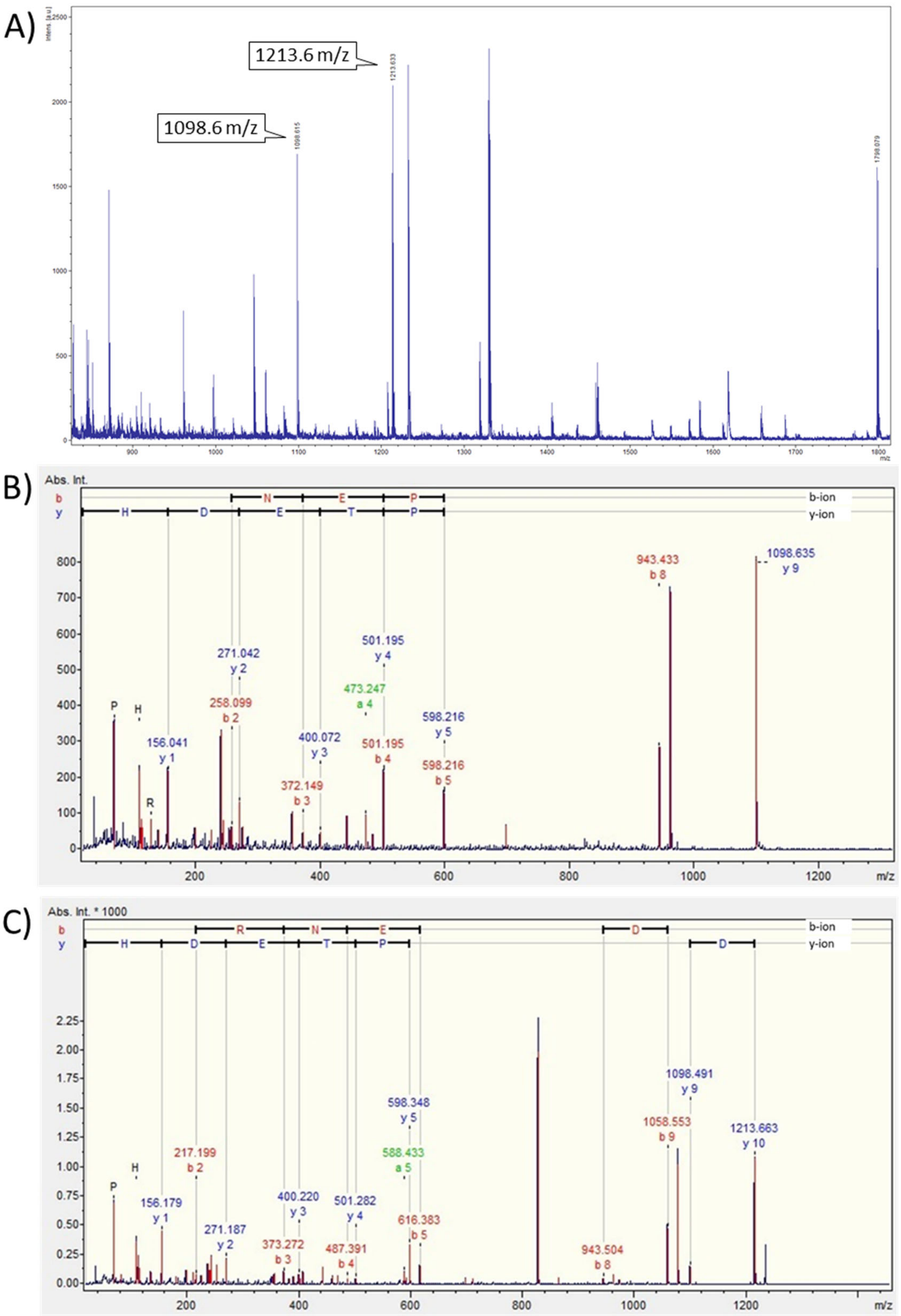


**Figure S3.** MS spectrum of C3-5 (**A**) and MS/MS identification of two peaks of *m*/*z* 1098.615 (**B**) and 1213.633 (**C**).

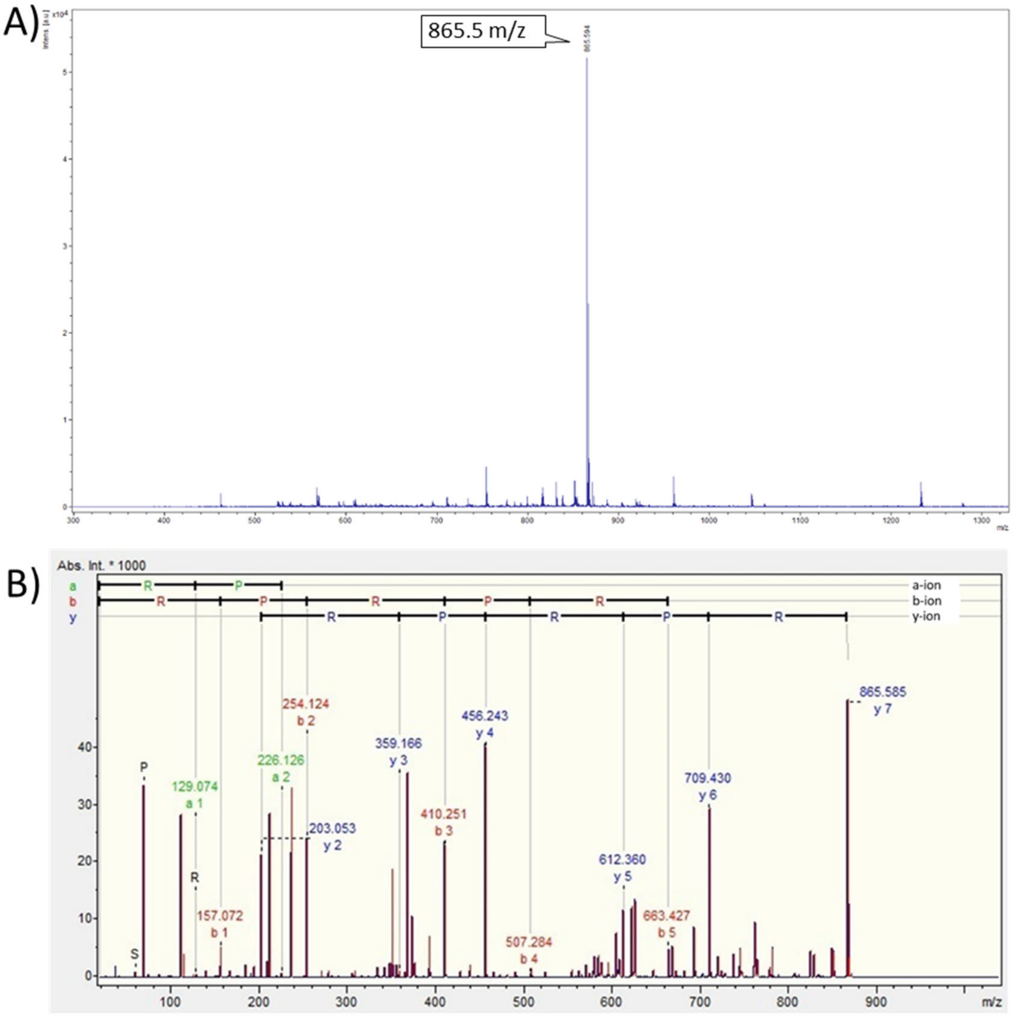


**Figure S4.** MS spectrum of C3-6 (**A**) and MS/MS identification of a peak of *m*/*z* 865.548 (**B**).

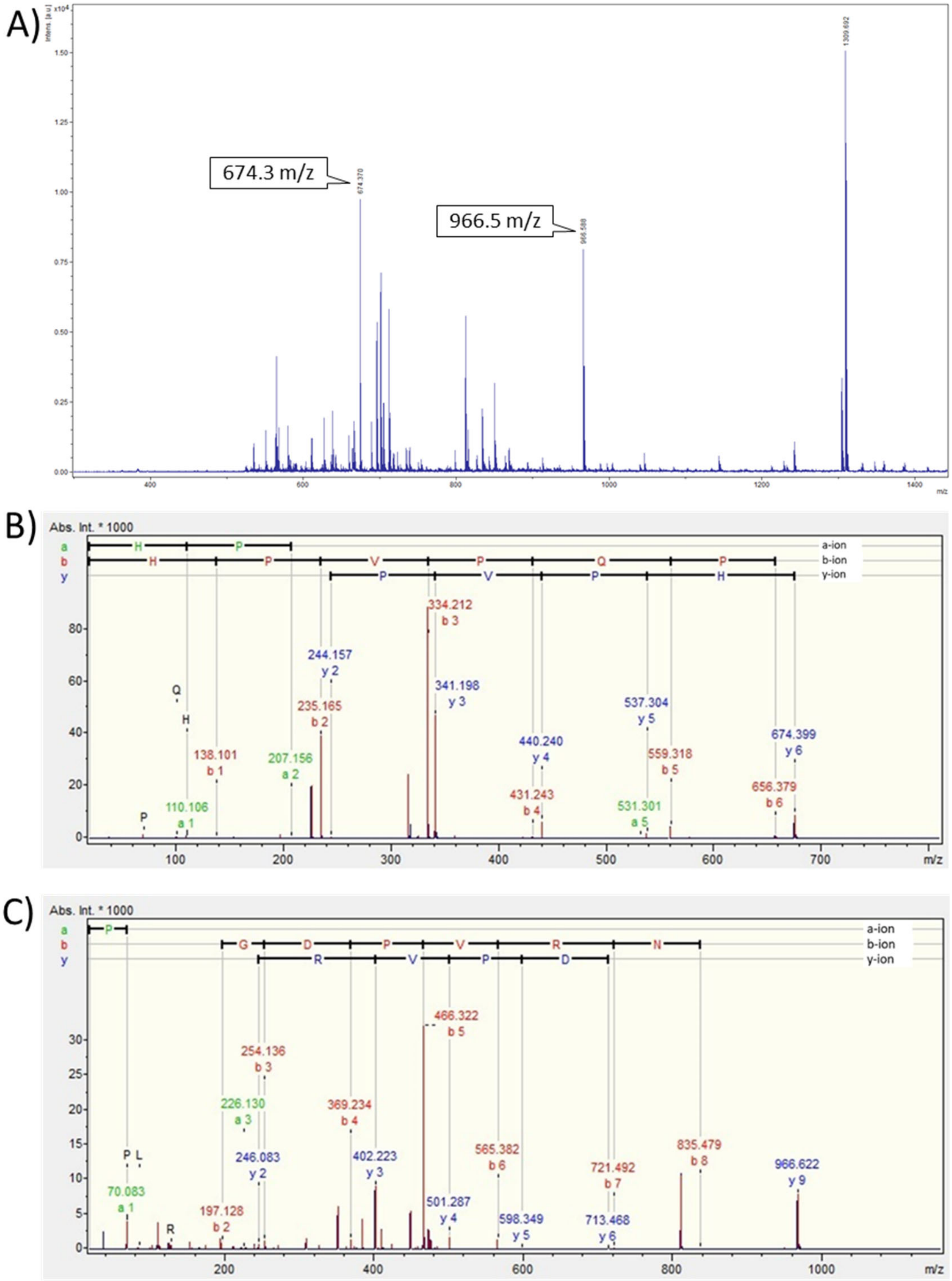


**Figure S5.** MS spectrum of C3-8 (**A**) and MS/MS identification of two peaks of *m/z* 674.370 (**B**) and 966.588 (**C**).

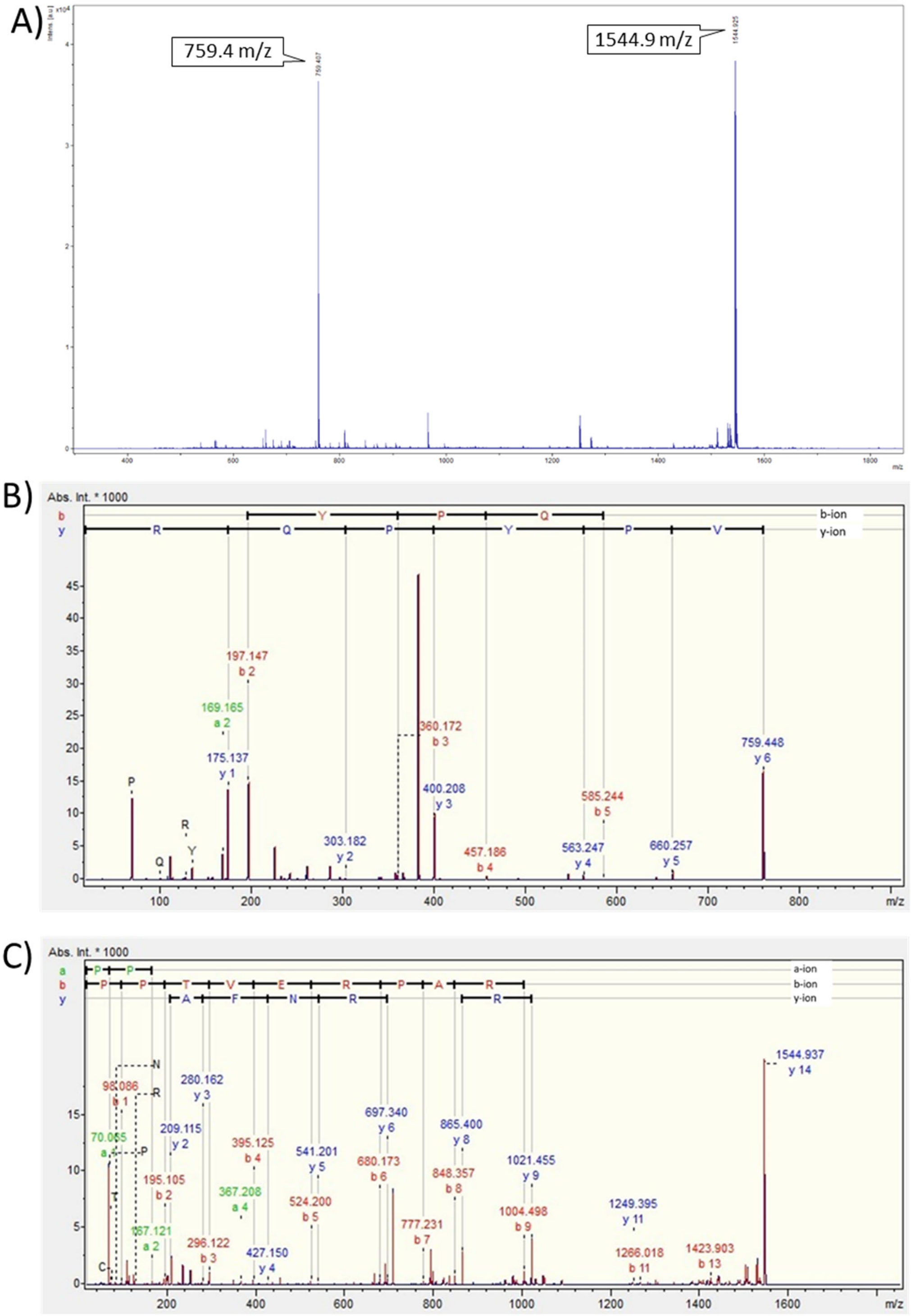


**Figure S6.** MS spectrum of C3-9 (**A**) and MS/MS identification of two peaks of *m*/*z* 759.415 (**B**) and 1544.925 (**C**).

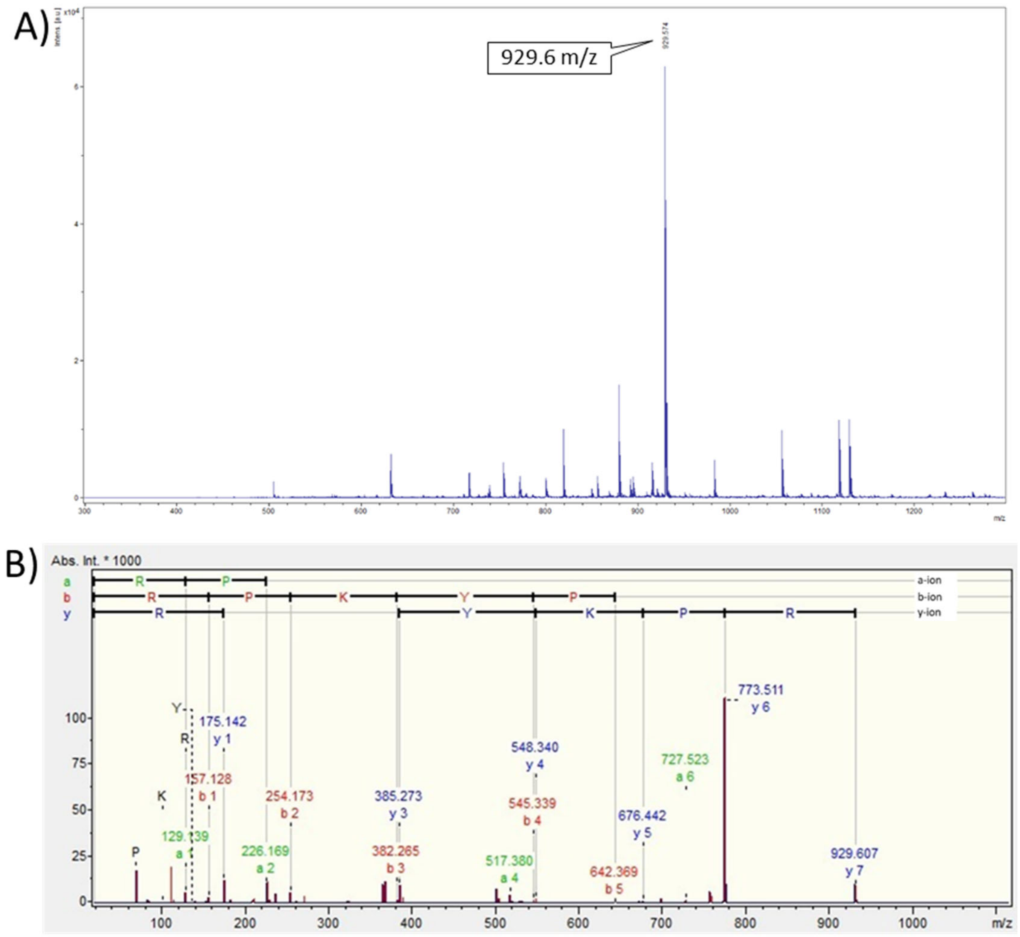


**Figure S7.** MS spectrum of C3-10 (**A**) and MS/MS identification of a peak of *m*/*z* 929.574 (**B**).

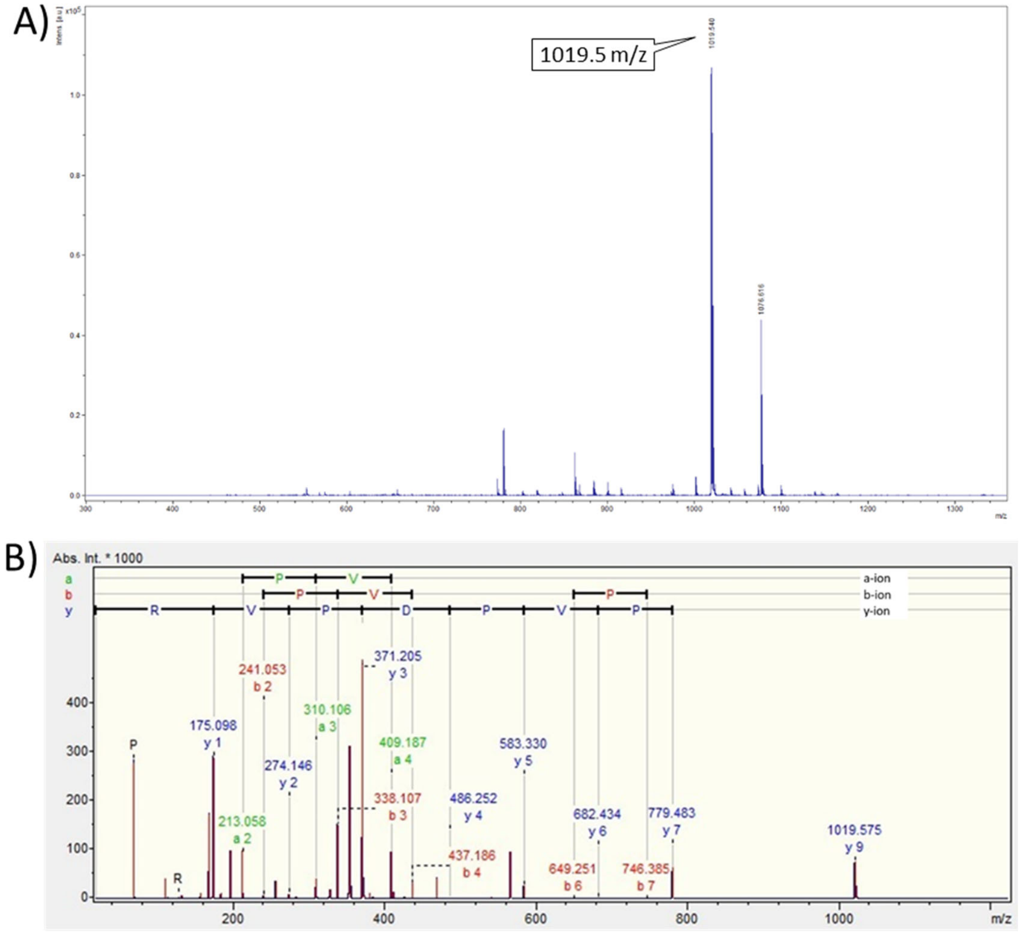


**Figure S8.** MS spectrum of C3-11 (**A**) and MS/MS identification of a peak of *m*/*z* 1019.540 (**B**).

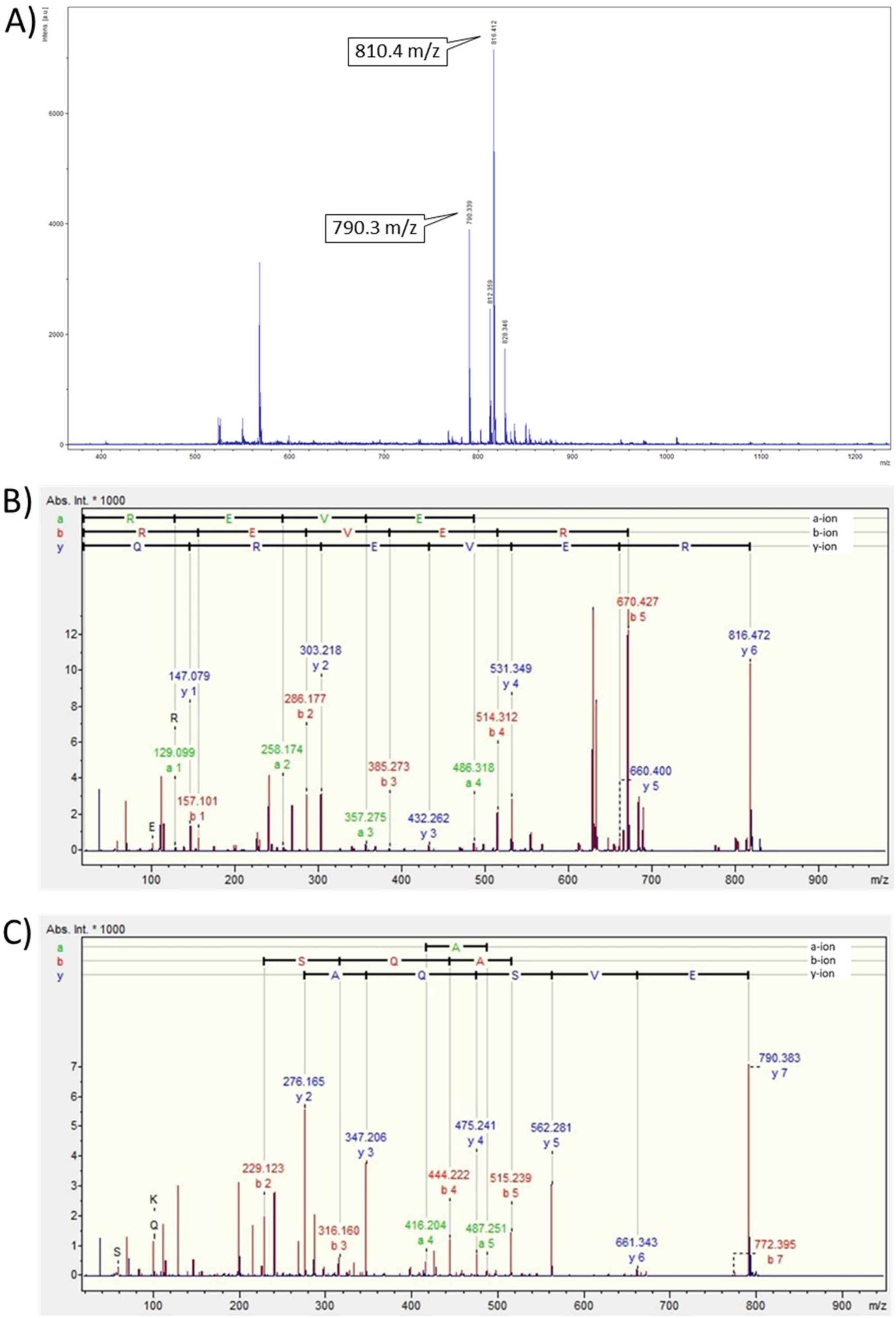


**Figure S9.** MS spectrum of H1-1 (**A**) and MS/MS identification of two peaks of *m*/*z* 816.432 (**B**) and 790.394 (**C**).

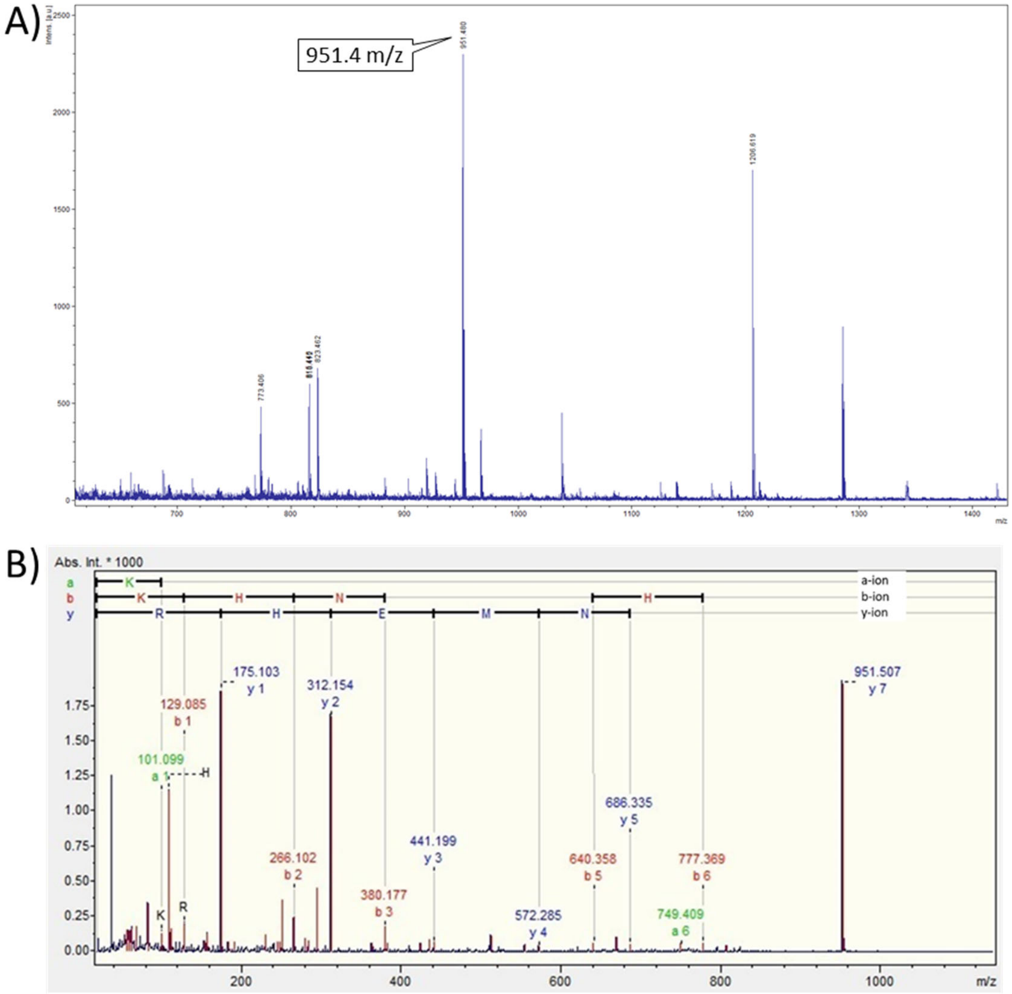


**Figure S10.** MS spectrum of H1-2 (**A**) and MS/MS identification of a peak of *m*/*z* 951.458 (**B**).

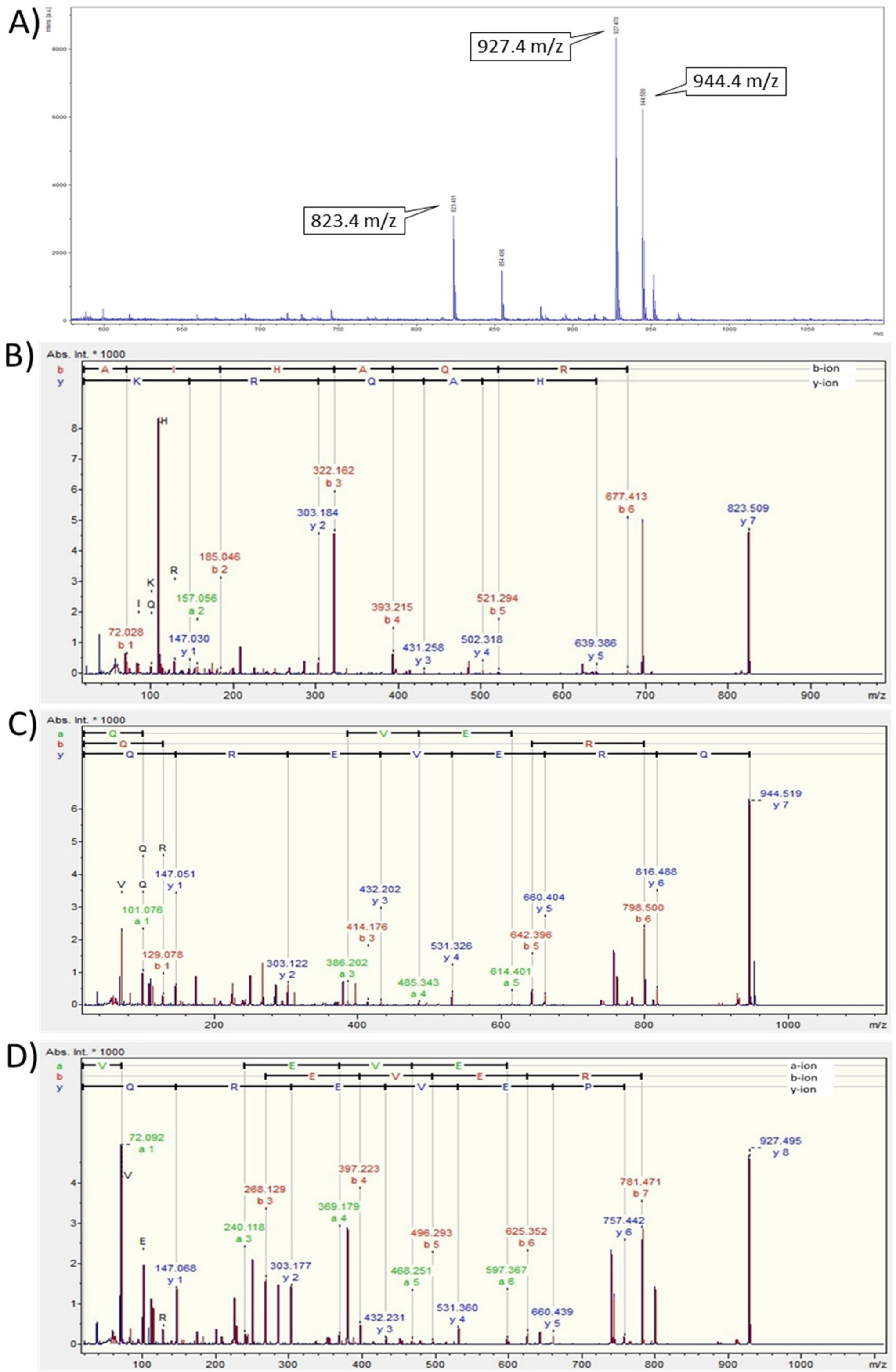


**Figure S11.** MS spectrum of H1-3 (**A**) and MS/MS identification of tree peaks of *m*/*z* 823.490 (**B**), 944.491 (**C**), and 927.470 (**D**).

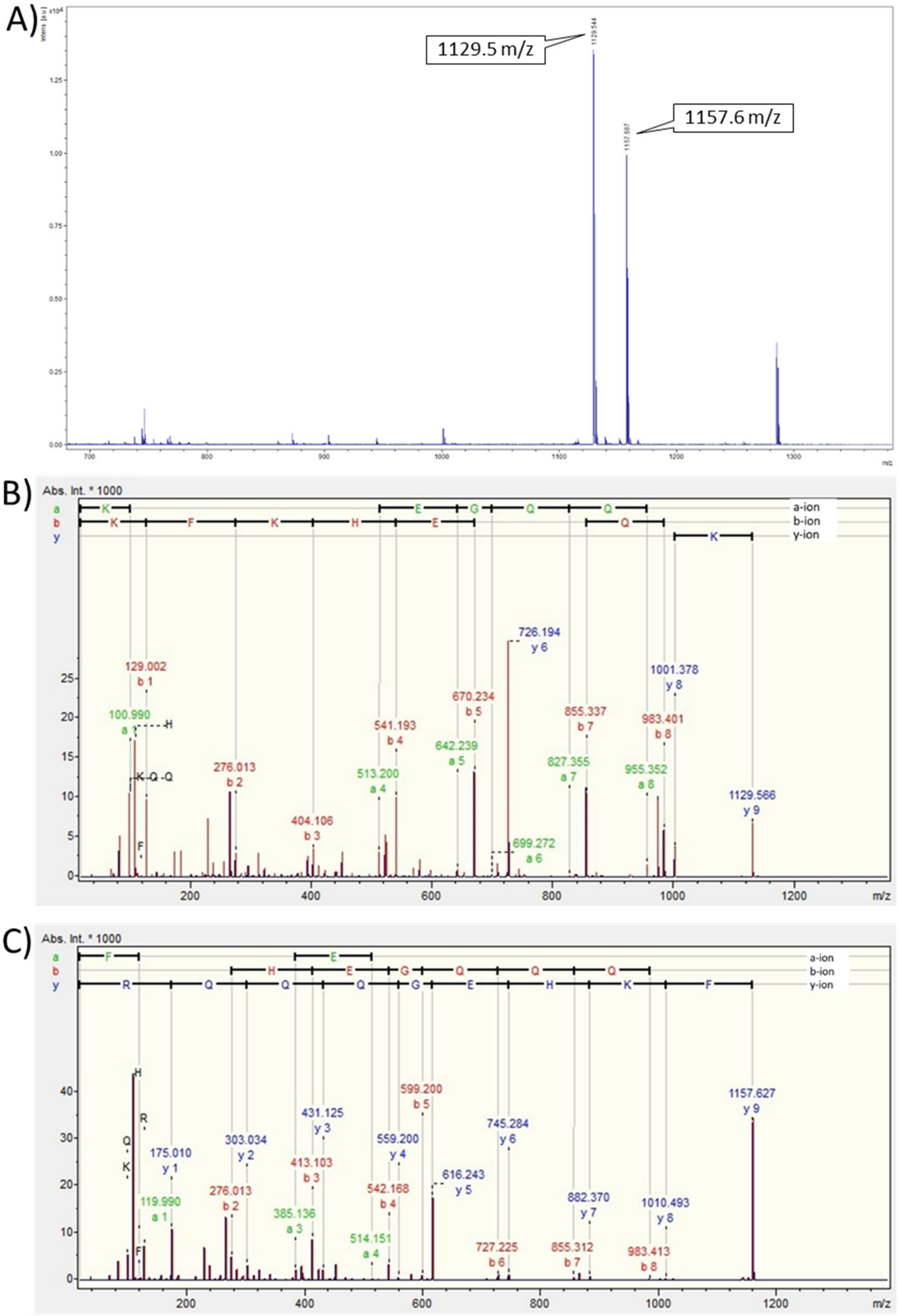


**Figure S12.** MS spectrum of H1-4 (**A**) and MS/MS identification of two peaks of *m/z* 1129.575 (**B**) and 1157.581 (**C**).

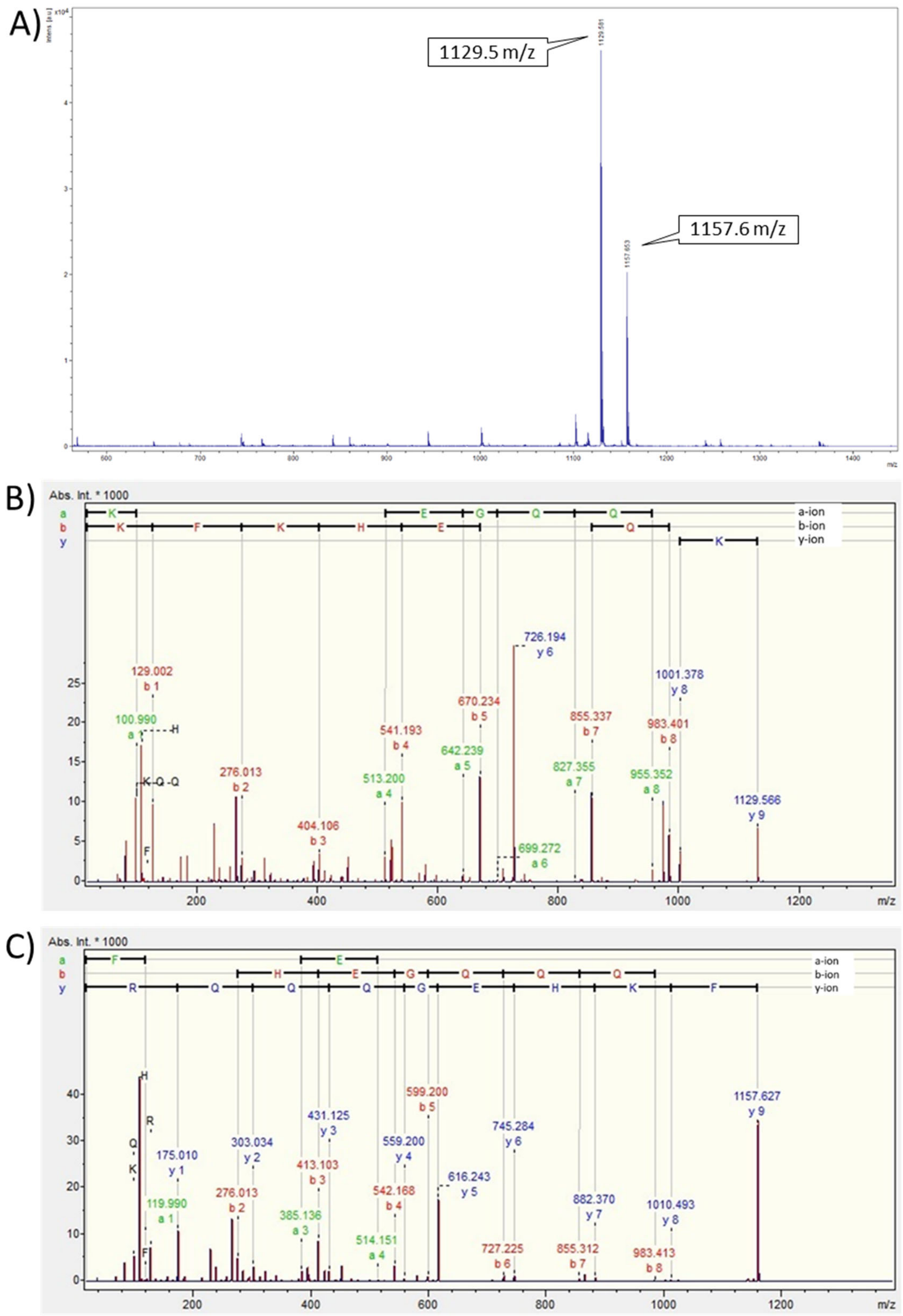


**Figure S13.** MS spectrum of H1-6 (**A**) and MS/MS identification of two peaks of *m/z* 1129.575 (**B**) and 1157.581 (**C**).

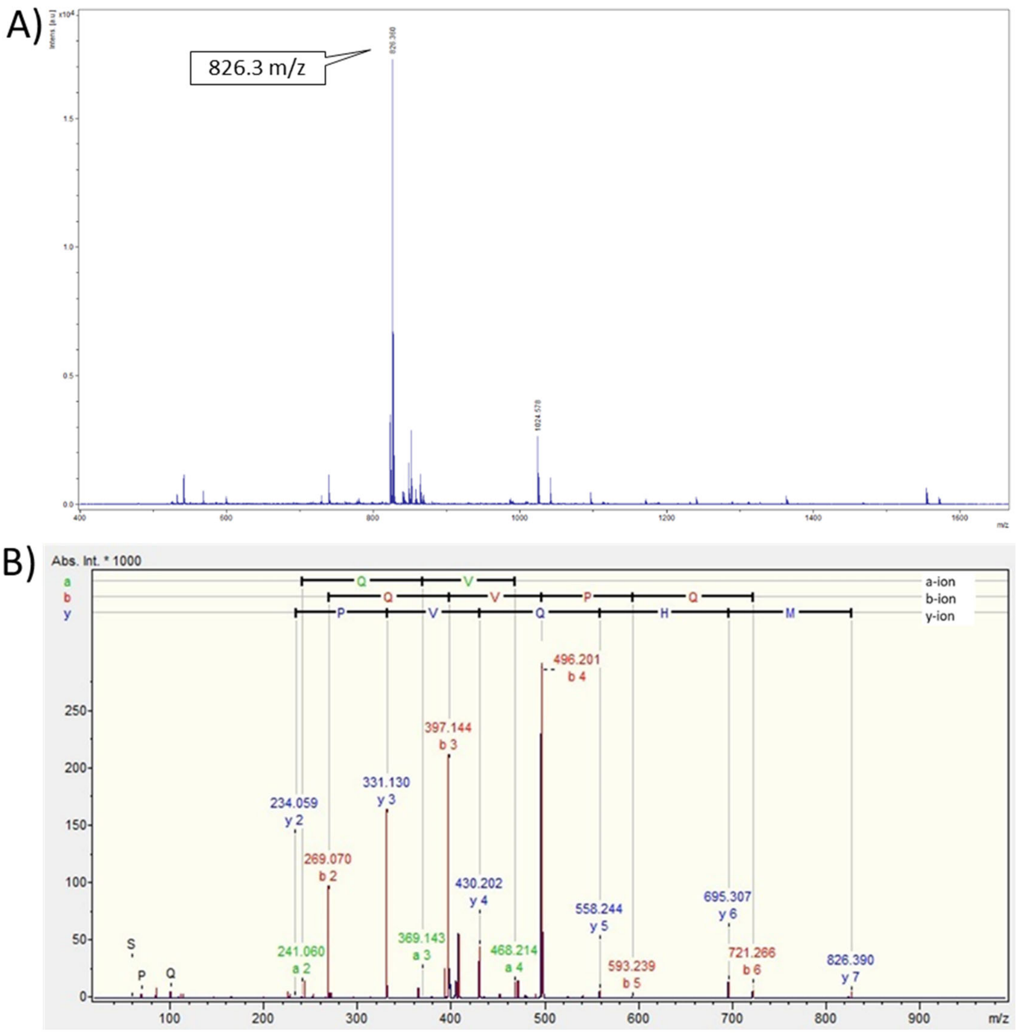


**Figure S14.** MS spectrum of H1-7 (**A**) and MS/MS identification of a peak of *m*/*z* 826.388 (**B**).

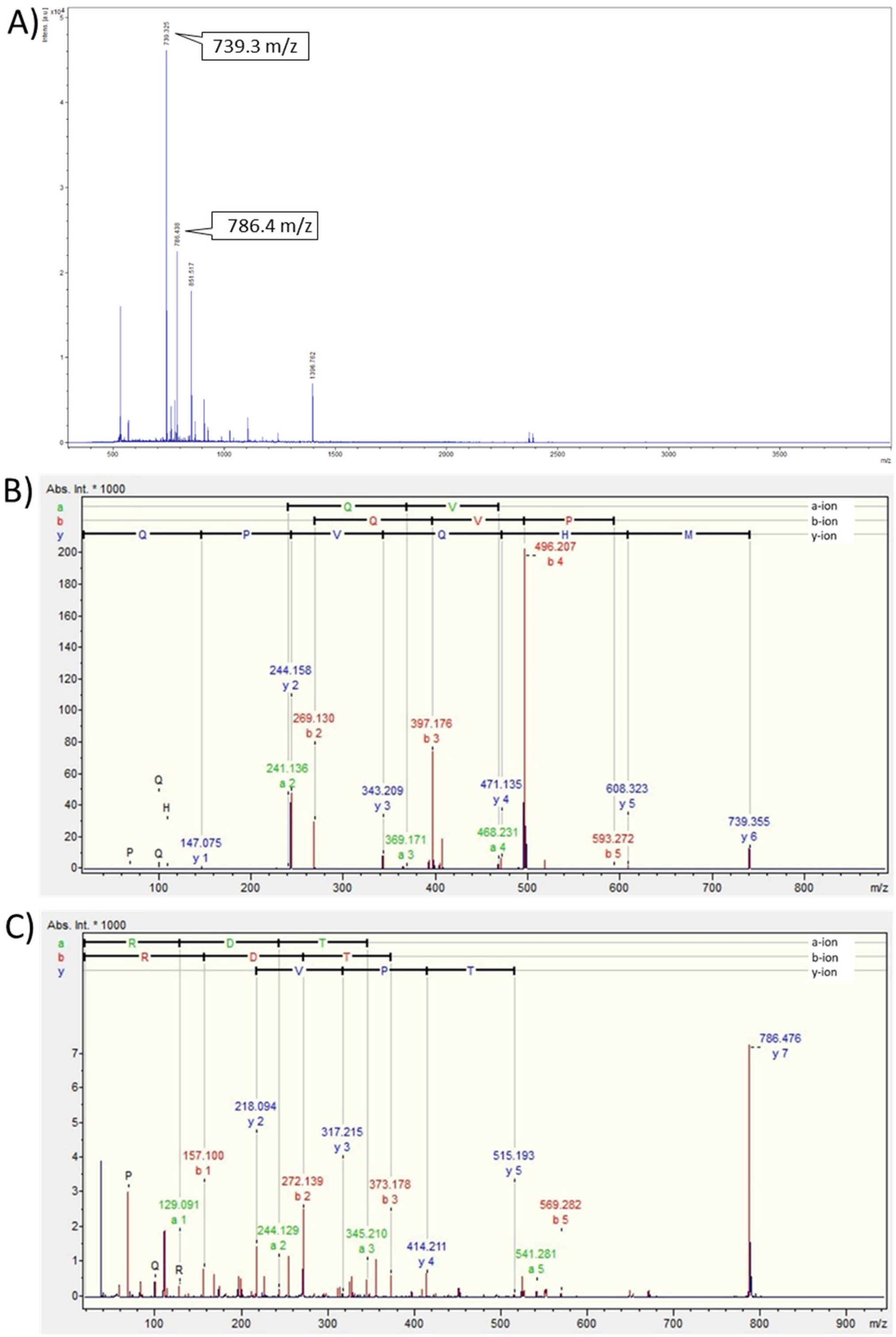


**Figure S15.** MS spectrum of H1-8 (**A**) and MS/MS identification of two peaks of *m*/*z* 739.356 (**B**) and 786.410 (**C**).

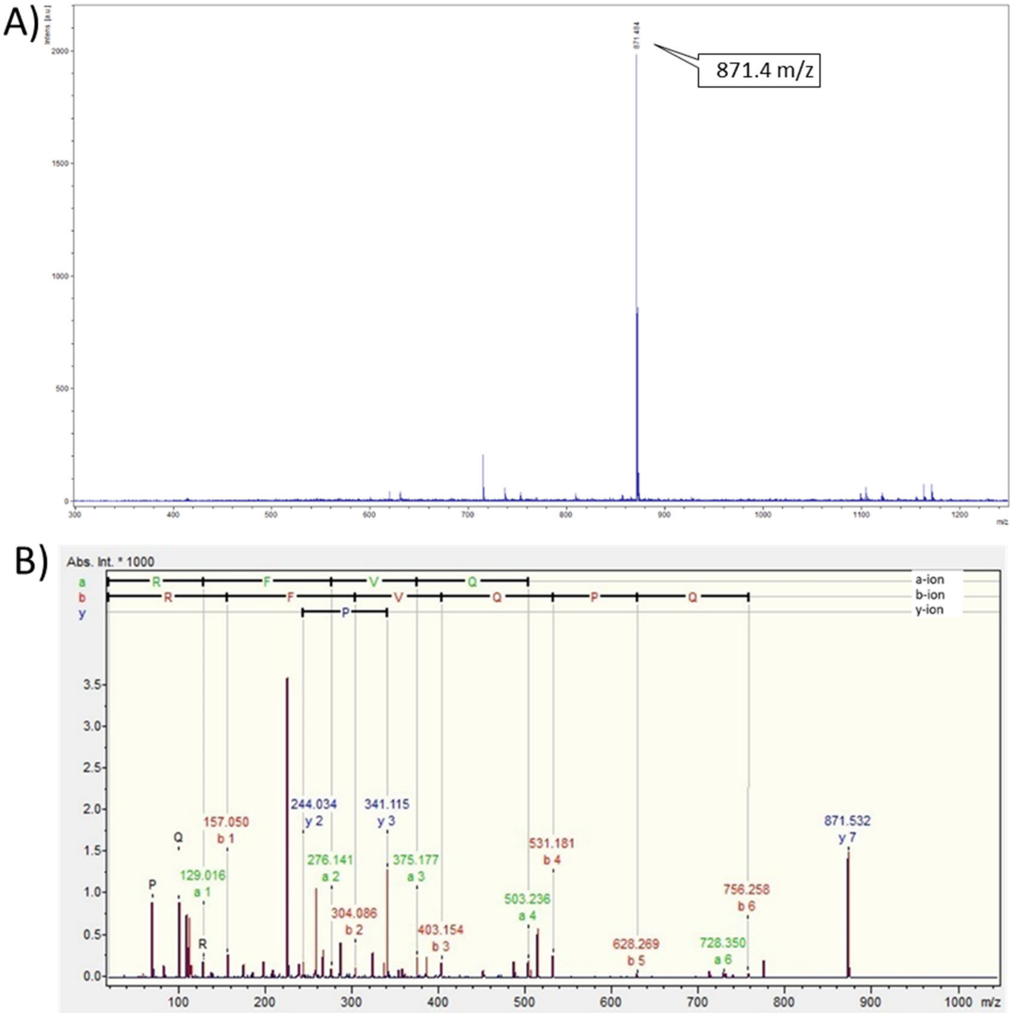


**Figure S16.** MS spectrum of H1-10 (**A**) and MS/MS identification of a peak of *m*/*z* 871.484 (**B**).

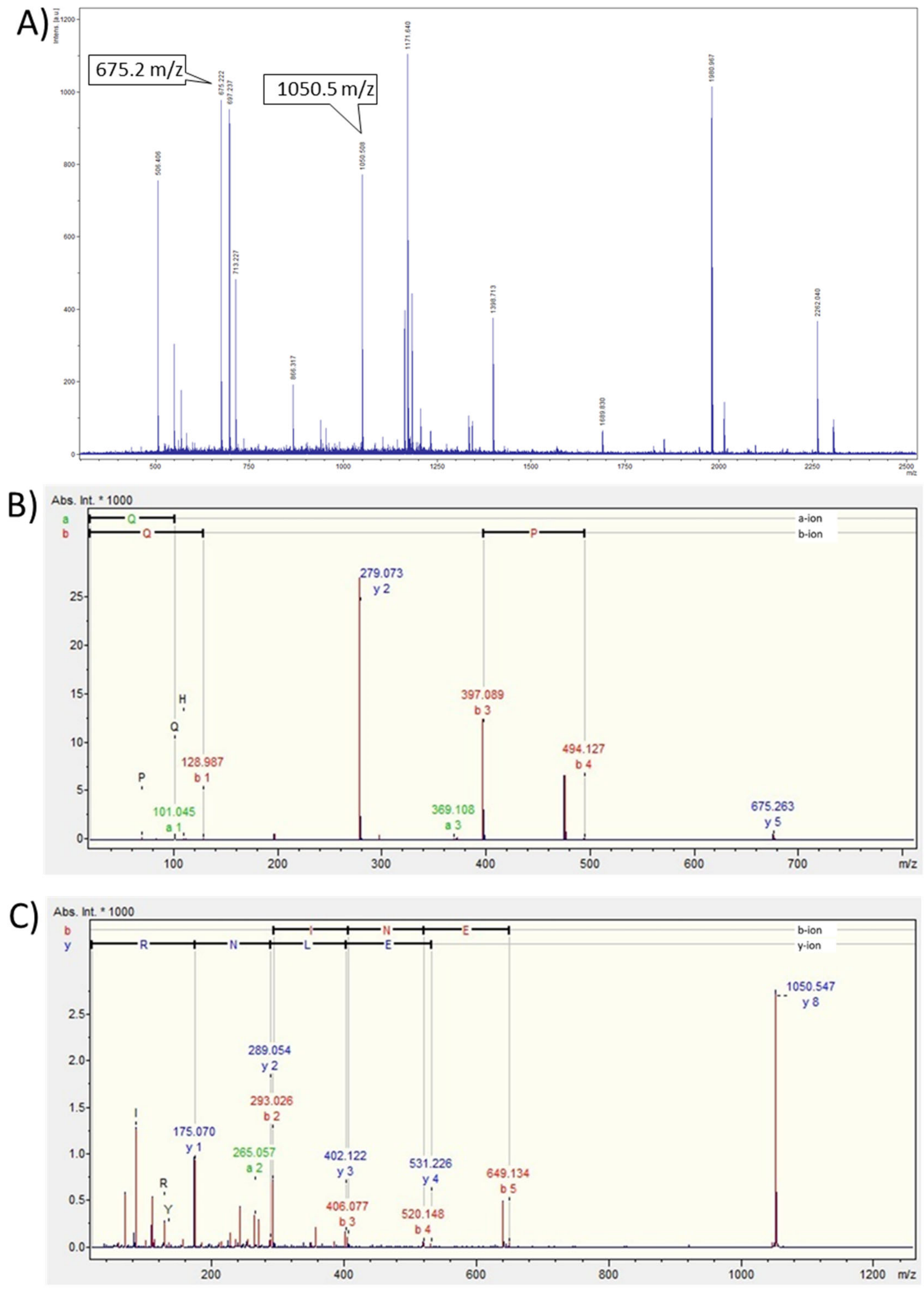


**Figure S17.** MS spectrum of H1-13 (**A**) and MS/MS identification of two peaks of *m*/*z* 675.222 (**B**) and 1050.508 (**C**).

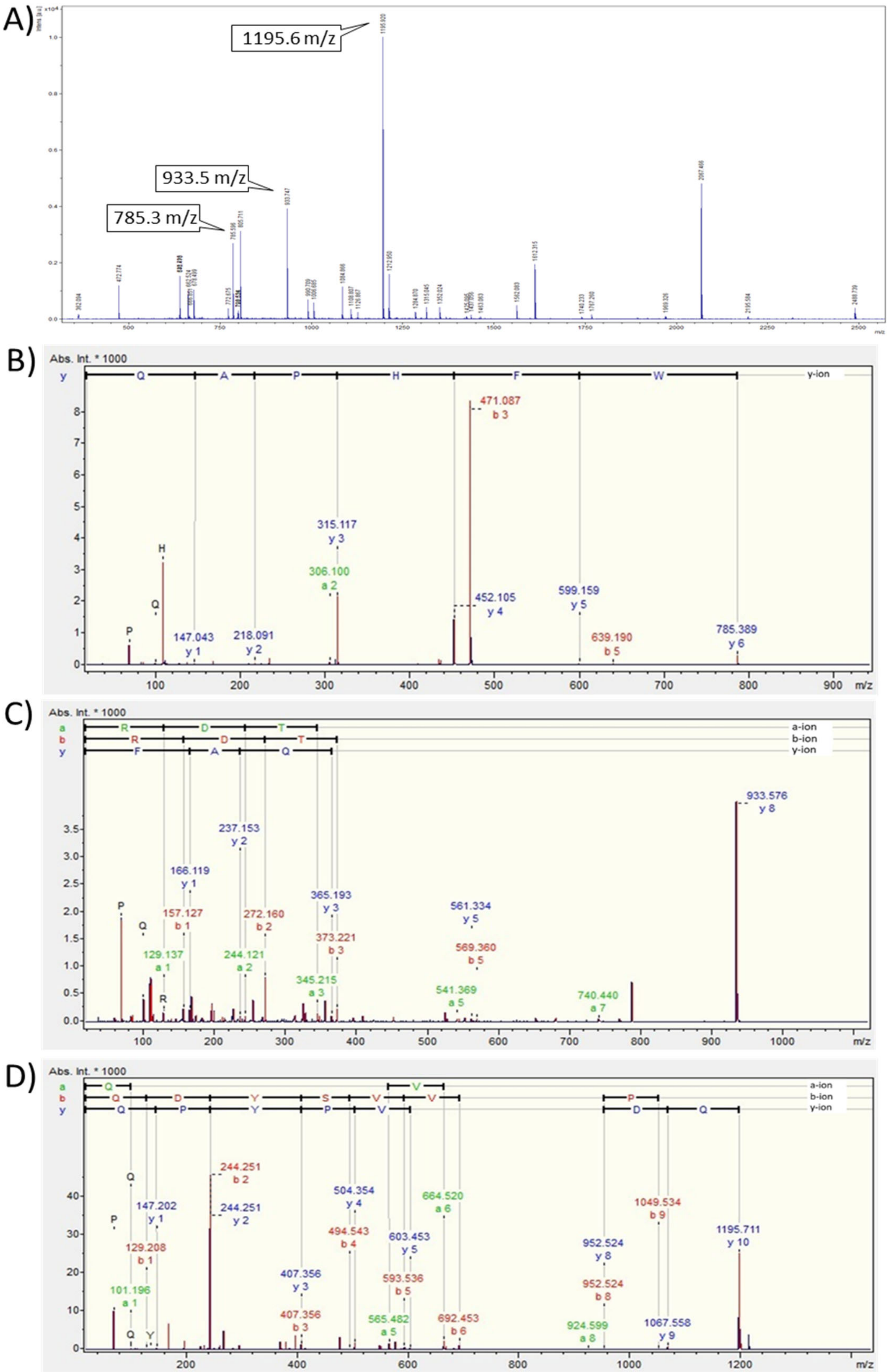


**Figure S18.** MS spectrum of H1-14 (**A**) and MS/MS identification of three peaks of *m*/*z* 785.358 (**B**), 933.512 (**C**), and 1195.652 (**D**).

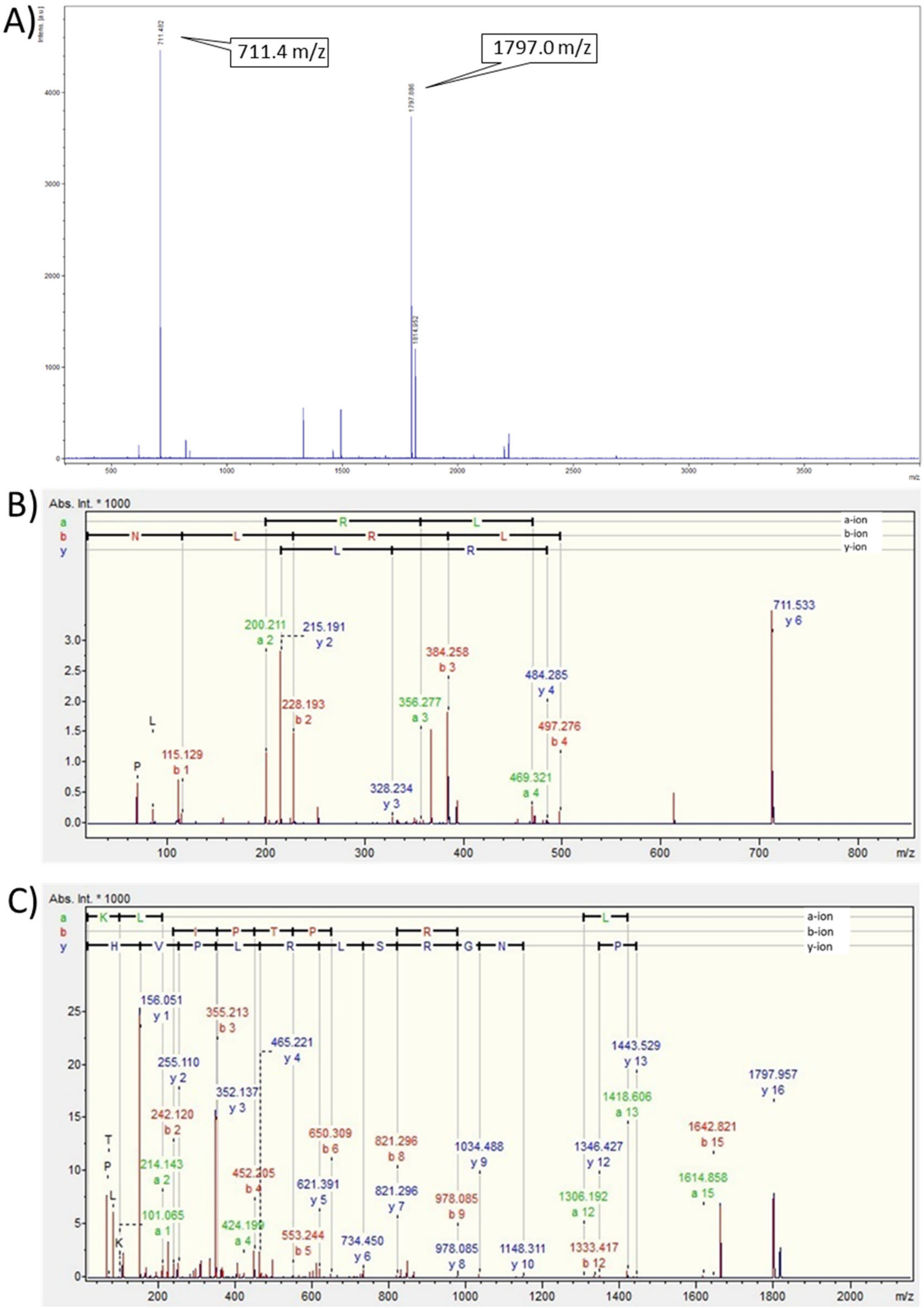

**Figure S19.** MS spectrum of H1-19 (**A**) and MS/MS identification of two peaks of *m*/*z* 711.451 (**B**) and 1797.000 (**C**).

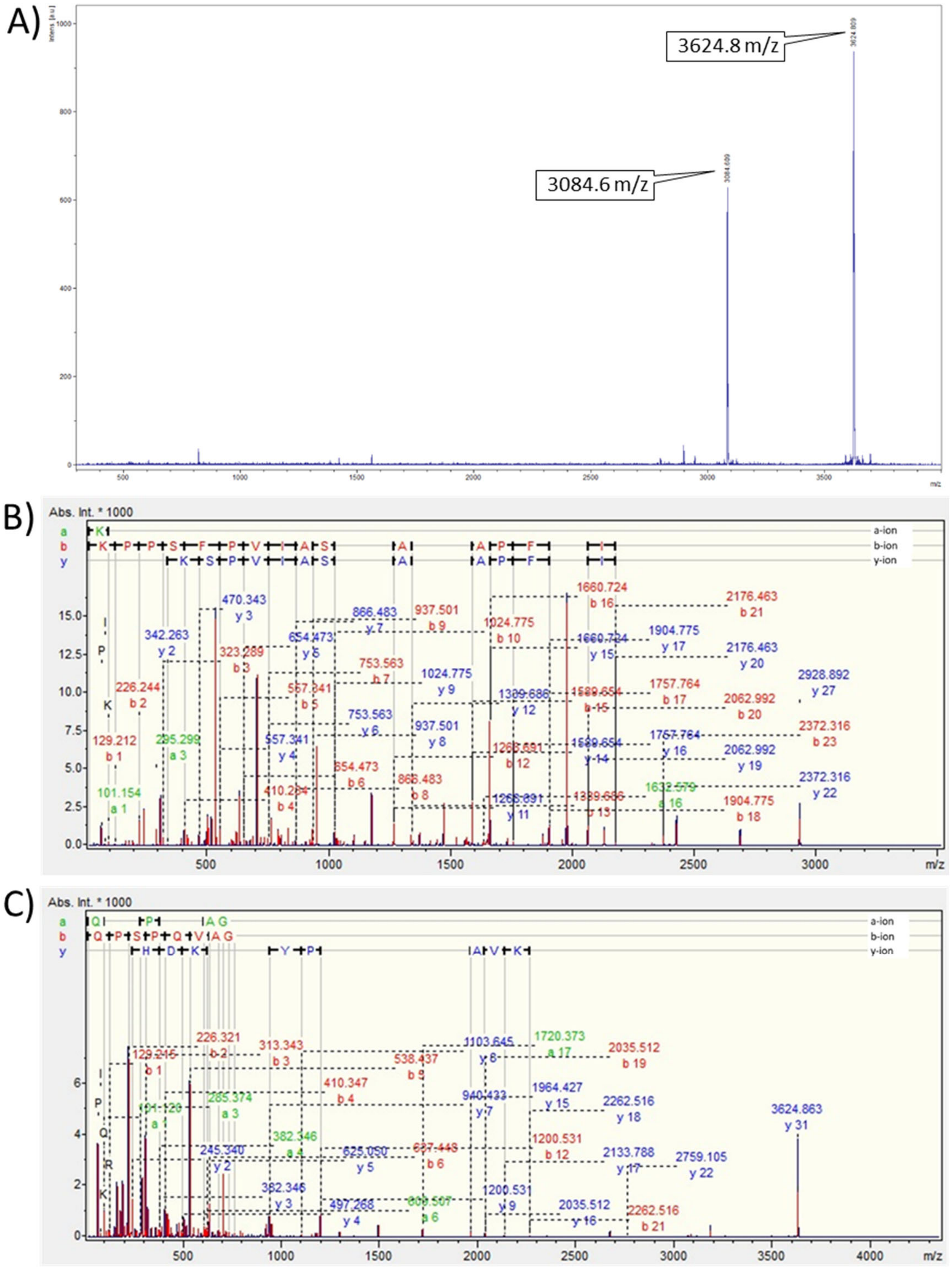

**Figure S20.** MS spectrum of H1-20 (**A**) and MS/MS identification of two peaks of *m/z* 3084.609 (**B**) and 3624.809 (**C**).

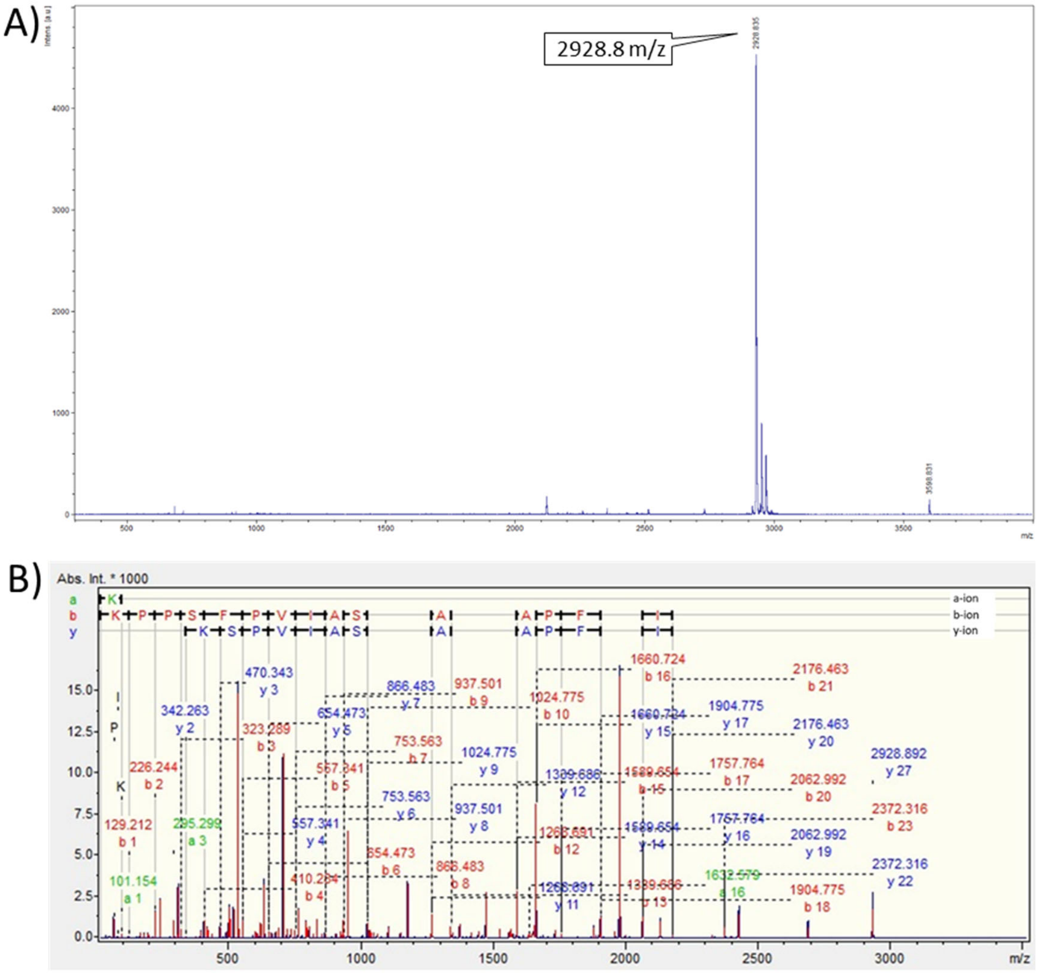


**Figure S21.** MS spectrum of H1-21 (**A**) and MS/MS identification of a peak of *m*/*z* 2928.835 (**B**).

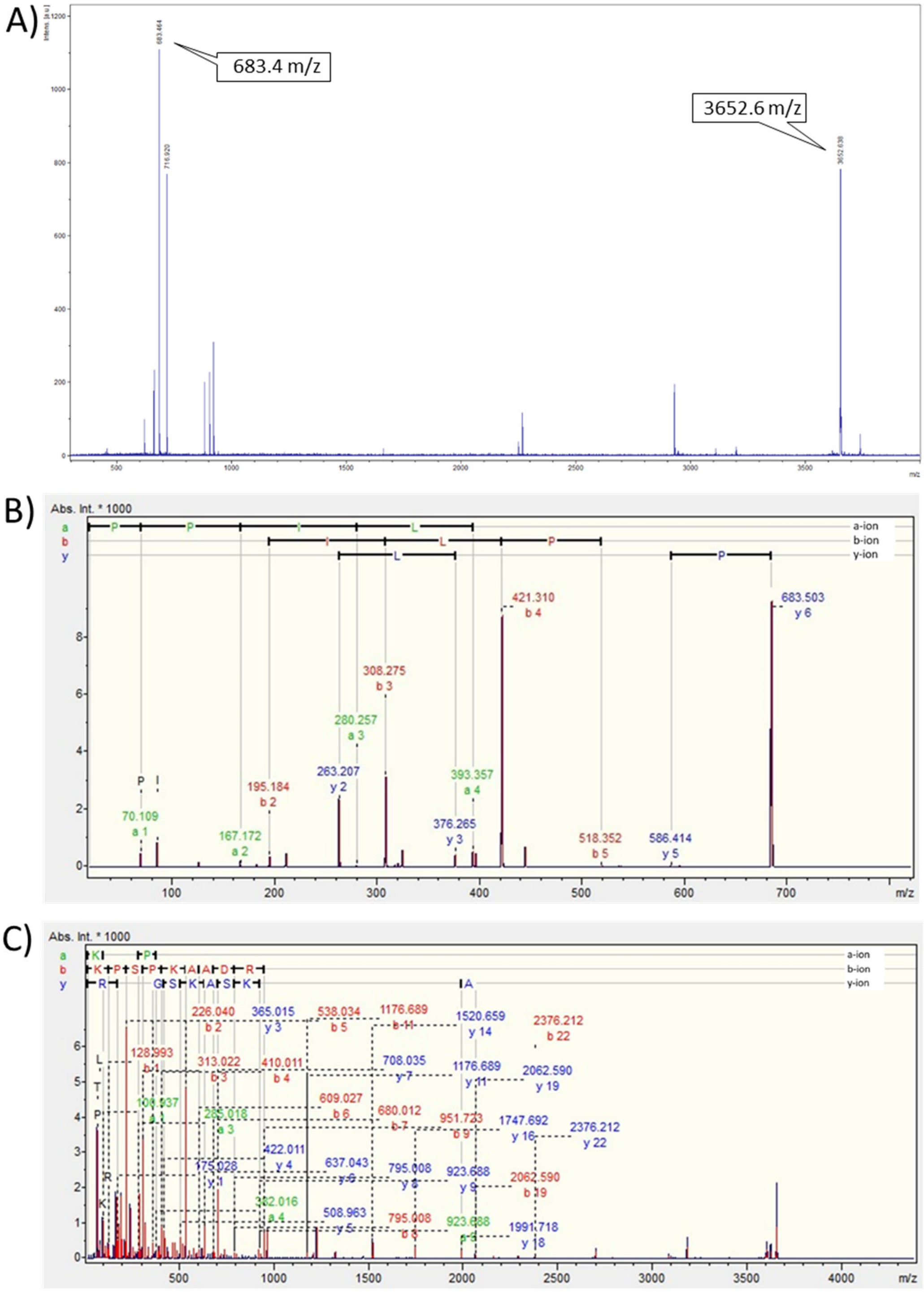


**Figure S22.** MS spectrum of H1-22 (**A**) and MS/MS identification of two peaks of *m*/*z* 683.468 (**B**) and 3652.638 (**C**).
